# Oarfish: enhanced probabilistic modeling leads to improved accuracy in long read transcriptome quantification

**DOI:** 10.1093/bioinformatics/btaf240

**Published:** 2025-07-15

**Authors:** Zahra Zare Jousheghani, Noor Pratap Singh, Rob Patro

**Affiliations:** Department of Electrical and Computer Engineering, University of Maryland, College Park, MD 20742, United States; Department of Computer Science, University of Maryland, College Park, MD 20742, United States; Department of Computer Science, University of Maryland, College Park, MD 20742, United States

## Abstract

**Motivation:**

Long-read sequencing technology is becoming an increasingly indispensable tool in genomic and transcriptomic analysis. In transcriptomics in particular, long reads offer the possibility of sequencing full-length isoforms, which can vastly simplify the identification of novel transcripts and transcript quantification. However, despite this promise, the focus of much long-read method development to date has been on transcript identification, with comparatively little attention paid to quantification. Yet, due to differences in the underlying protocols and technologies, lower throughput (i.e. fewer reads sequenced per sample compared to short-read technologies), as well as technical artifacts, long-read quantification remains a challenge, motivating the continued development and assessment of quantification methods tailored to this increasingly prevalent type of data.

**Results:**

We introduce a new method and corresponding user-friendly software tool for long-read transcript quantification called oarfish. Our model incorporates a novel coverage score, which affects the conditional probability of fragment assignment in the underlying probabilistic model. We demonstrate, in both simulated and experimental data, that by accounting for this coverage information, oarfish is able to produce more accurate quantification estimates than existing long-read quantification tools.

**Availability and Implementation:**

oarfish is implemented in the Rust programming language and is made available as free and open-source software under the BSD 3-clause license. The source code is available at https://www.github.com/COMBINE-lab/oarfish.

## 1 Introduction

Since the introduction of high-throughput RNA-sequencing (Bainbridge *et al.* 2006, Lister *et al.* 2008, Mortazavi *et al.* 2008, Nagalakshmi *et al.* 2008), the bioinformatics community has invested tremendous effort in the development of methods and software to tackle various challenges related to the analysis of this data. One of the first, and therefore one of the most fundamental challenges, is the accurate quantification of transcript and gene expression from this sequencing data, which has spurred the development of many methods for transcript quantification [e.g. (Nicolae *et al.* 2011, Li and Dewey 2011, Turro *et al.* 2011, Glaus *et al.* 2012, Nariai *et al.* 2013, Patro *et al.* 2014, Hensman *et al.* 2015, Bray *et al.* 2016, Patro *et al.* 2017) among many others].

The majority of these tools focus on improving the accuracy, efficiency, or both, of quantification from high-throughput short-read data. While short reads offer high base-level accuracy, reproducibility, and throughput, their main drawback is their short length (usually <350bp), much shorter than the average length of spliced human mRNA transcripts [estimated at ∼2 kb (Lewin and Dover 1994)]. This mismatch creates ambiguity in determining the exact origin of many reads, a challenge known as fragment or read-to-transcript ambiguity (Baldoni *et al.* 2024). This ambiguity leads to uncertainty in transcript abundance estimates, complicating downstream analyses (Pimentel *et al.* 2017, Zhu *et al.* 2019). Although techniques like paired-end sequencing (Liu *et al.* 2016, Baldoni *et al.* 2024) help reduce this uncertainty, it remains a fundamental limitation of short-read technologies.

While the methods developed for short-read transcript quantification, e.g. (Nicolae *et al.* 2011, Li and Dewey 2011, Turro *et al.* 2011, Glaus *et al.* 2012, Nariai *et al.* 2013, Patro *et al.* 2014, Hensman *et al.* 2015, Bray *et al.* 2016, Patro *et al.* 2017) have led to improved quantification accuracy and computational efficiency, they cannot overcome limitations inherent in the sequencing technologies themselves. However, the advent of long-read sequencing (and its continued developments in terms of reduced error rates and improved throughput) promises to mitigate these limitations and transform transcriptome analysis, much like it has done with genome assembly (Nurk *et al.* 2022). Long reads can capture full-length transcripts in a single read, eliminating, in many cases, ambiguity about transcript origin, with significant implications for both transcript discovery and quantification. Yet, long reads bring their own challenges compared to short reads, higher error rates (in some technologies) and reduced sequencing depth. Thus, even when sequencing a similar total number of nucleotides as a short-read sample, long reads comprise more nucleotides *per-read*, and so typically result in fewer independent measurements.

Currently, there are two major long read sequencing technologies; Oxford Nanopore Technologies (ONT) (Wang *et al.* 2021, Wongsurawat *et al.* 2022, ONT 2024) and Pacific Biosciences (PacBio) (Rhoads and Au 2015, Wenger *et al.* 2019, Pac 2024), each technology with its own benefits and drawbacks. While the PacBio long reads have high accuracy and low error rate (especially HiFi reads), they typically have lower throughput and higher cost than ONT sequencing, though throughput can be improved substantially using techniques such as MAS-ISO-seq (and Kinnex) (Al’Khafaji *et al.* 2023). ONT sequencing typically provides higher throughput and can therefore be more cost-efficient, but the generated reads typically have higher error rates. Other differences in capabilities arise from the actual mechanisms by which sequencing is performed—for example, ONT sequencing is capable of sequencing either cDNA (as is traditionally used in RNA-seq) or performing direct RNA sequencing without first requiring reverse transcription, which also permits direct detection of base-level modifications of the underlying RNA molecule.

Long-read RNA sequencing is not without a growing collection of associated quantification methods. In response to the limitations of traditional tools, methods like TALON (Wyman *et al.* 2019), FLAIR (Tang *et al.* 2020), and Mandalorion (Byrne *et al.* 2017) have emerged, and while these tools support quantification, they primarily focus on novel transcript identification. For quantification, some tools initially developed for short-read data, like salmon (Patro et al. 2017) have since been augmented to support long-read data by adjusting the sequencing error models appropriately and removing the length dependence in the underlying graphical model, and recent benchmarks have shown that this approach can work well for long read quantification (Chen *et al.* 2023). Likewise, Kallisto was recently augmented to enable long-read quantification by modifying the pseudoalignment approach and altering the effective transcript length used during quantification (Loving *et al.* 2025). At the same time, many tools have been developed specifically for use with long-read data, such as Bambu (Chen *et al.* 2023), ESPRESSO (Gao *et al.* 2023), IsoQuant (Prjibelski *et al.* 2023), NanoCount (Gleeson *et al.* 2022), and TranSigner (Ji and Pertea 2024), among others. These tools differ in their approaches to probabilistic read allocation. Bambu uses EM with transcript categories; ESPRESSO relies on a compatibility-based latent model; NanoCount considers alignment counts and applies filters; TranSigner uses EM with a read-focused “drop” algorithm to improve estimates.

In this work, we develop a new method for transcript quantification using long reads, and implement this approach in an efficient and easy-to-use tool called oarfish. Compared to existing methods, oarfish includes a novel coverage model term and adopts distinct approaches for modeling key parts of the data (e.g. a new alignment scoring model). We focus on the quantification of both ONT and PacBio long reads. The models we develop and implement are likely largely technology agnostic, and therefore are likely also to increase the accuracy of abundance estimated generated using newly developed long-read technologies (Zhang *et al.* 2024). Likewise, reduced sequencing error and more accurate reads, if obtained at the same sequencing depth, are certainly key factors that we expect to contribute to more accurate quantification results and, subsequently, more accurate and precise transcriptome analysis.

## 2 Background

### 2.1 A generative model for RNA-seq data

In Li *et al.* (2010), the authors propose a generative model for RNA-seq sequencing, which has also been adopted by many subsequent tools for short-read quantification. A plate diagram of this generative model is provided in Fig. 1 of Li *et al.* (2010), and an extended model is presented in Fig. 4 of Li and Dewey (2011). The corresponding likelihood of a given collection of data (i.e. sequenced fragments and their corresponding alignments) is given as follows:


(1) $$L(R,\theta )\propto P(R|\theta )=\prod_{n=1}^{N}\sum_{i=0}^{M}\theta_{i}P(r_{n}|G_{n}=i).$$

The likelihood defined in Equation (1) can be maximized (locally) using an Expectation-Maximization (EM) (Dempster *et al.* 1977) algorithm, which the authors derive for this particular likelihood in (Li *et al.* 2010, Li and Dewey 2011). This algorithm is then used to obtain a maximum likelihood estimate (MLE) of the model’s parameters $\theta =\left[\theta_{0},\theta_{1},\ldots ,\theta_{M}\right]$, which correspond to the relative abundances of the isoforms, given the observed data $R=\left[r_{1},r_{2},\ldots ,r_{N}\right]$. Here M is the number of isoforms and N is the number of fragments. In Equation (1), i=0 represents a “noise” isoform, and variables $\theta_{i},r_{n},G_{n}$ denote the relative abundance of $i^{\text{th}}$ isoform, the $n^{\text{th}}$ fragment, and the (latent) random variable assigning the $n^{\text{th}}$ fragment to the $i^{\text{th}}$ isoform. Therefore, $P(r_{n}|G_{n}=i)$ is the conditional probability of observing the fragment $r_{n}$ given that it arises from the $i^{\text{th}}$ isoform.

This quantification model, or variants of it, have been used in many different methods designed for isoform expression quantification using short read RNA-seq data (Li and Dewey 2011, Nariai *et al.* 2013, Roberts and Pachter 2013) (and many others).

Shortly after Li *et al.* introduced RSEM (Li *et al.* 2010), Turro *et al.* (2011) introduced MMSeq. The latter tool is particularly notable because it introduced a related but distinct model in which likelihood was no longer evaluated (and parameters no longer optimized) at the level of individual fragments, but rather at the level of *equivalence classes* of fragments, where two fragments $r_{i}$ and $r_{j}$ are considered equivalent if and only if they map or align to the same set of transcript targets. By working with such equivalence classes, and retaining their multiplicity (i.e. the number of fragments belonging to each such class), MMSeq defined a similar but simplified likelihood which is *much* faster to optimize, as the E step of the EM algorithm scales with the number of distinct equivalence classes rather than with the number of distinct fragments. This alternative formulation of the problem represents an *approximate* factorization of the likelihood function, and has also since been used in several transcript quantification tools (Turro *et al.* 2011, Patro *et al.* 2014, Zhang and Wang 2014, Bray *et al.* 2016, Patro *et al.* 2017). Between the extremes of the fragment-level model of Li *et al.* and the compatibility-based model of Turro *et al.*, there exists a range of potential factorizations of the likelihood function, in which one can trade off computational efficiency for model fidelity (Zakeri *et al.* 2017), and factorizations can be derived that improve quantification accuracy relative to the compatibility-based model while still retaining rapid quantification speed.

### 2.2 Long-read RNA-seq data and quantification models

If the long read RNA-seq data consist of reads that always span the whole length of isoforms from which they are sequenced, then isoform expression quantification is a reasonably straightforward task, since there will be comparatively few multi-mapped reads (i.e. only among those cases where one transcript is a proper subsequence of another). In such a case, the estimate for the relative abundance of an isoform is just the fraction of the sequenced reads that map to this isoform. However, although it is expected that the long reads have the same length as the isoforms sequenced (i.e. that the reads represent full-length transcripts), most of these reads are, in fact, shorter than the isoforms from which they originate due to technical artifacts, unexpected errors, fragmentation or breakage during sequencing, or for other reasons (Amarasinghe *et al.* 2020). Additionally, the sequenced reads may constitute the full length of the sequenced molecule, but may not match the full length of the annotated isoform, because of well-known biological processes, such as transcript degradation.

The length distribution model for the long-read RNA-seq dataset, sequenced from the Hct116 cell line (as discussed in Section D of the Supplementary), is presented along with the distribution of aligned lengths of the reads to the isoform and the distribution of isoform lengths in Fig. 3, available as supplementary data at *Bioinformatics* online. We observe that the typical length of isoforms is 1.5 times longer than the typical length of the aligned segments of the reads that map to these isoforms. Therefore, we also observe multimapped reads at a non-trivial rate of 70–80% in long-read RNA-seq alignments. As such, although probabilistic models and inference techniques developed for short-read data (Li *et al.* 2010) can be useful for long-read quantification, the generative model should be modified to reflect the fact that long-read RNA-seq experiments typically follow protocols that differ in important ways from those used in short-read sequencing.

One fundamental difference, on which we focus here, is that long-read RNA-seq protocols do not include systematic fragmentation prior to sequencing. Since the fragment length of short read protocols is much less than the typical length of the transcripts being sequenced, and since we wish to be able to generate reads from (nearly) any position within a transcript, these protocols almost universally contain a fragmentation step, in which the initial full-length transcripts are randomly fragmented, using one of several different techniques (Hess *et al.* 2020, Hu *et al.* 2021).

The fundamental effect of this fragmentation process on the generative model is that we expect the number of reads generated from a particular isoform to be proportional to the *product of* the number of copies of that isoform and the isoform’s length. For example, if two isoforms A and B were present in equal numbers in the underlying sample, but isoform A was *k* times longer than B, then we would expect, on average, to sequence approximately *k* times as many fragments from A as from B. This leads the number of reads generated from a particular isoform to have a fundamental dependence on the isoform’s length. However, fragmentation is typically not a step in long-read RNA-seq protocols, so we instead expect the number of reads arising from an isoform to be directly proportional to the number of copies of that isoform in the sample, and not, additionally, dependent on the isoform’s length. As we explain later, this seeming simplification to the model also eliminates a useful source of evidence when trying to determine the likely allocation of a multimapping read between sequence-similar transcripts of different lengths.

Several computational tools, such as (Gleeson *et al.* 2022, Chen *et al.* 2023, Gao *et al.* 2023, Prjibelski *et al.* 2023, Ji and Pertea 2024), have been developed to quantify long-read RNA-seq data, but as discussed in the Introduction, they typically remove the length effect without addressing the subsequent reduction it entails in the ability of the model to distinguish ambiguous fragments. Hence, we propose a model that removes the length effect of the short read model, and also replaces it with a new term that seeks to increase the uniformity of coverage under the read assignment probabilities.

## 3 Methods and materials

We refine the generative model introduced in (Li *et al.* 2010) to enhance its applicability and accuracy, specifically in the context of long-read sequencing. In this work, we adopt a fragment-level likelihood, rather than a compatibility-based or alternative approximate likelihood factorization. We will augment the model with an alignment-level term (a modification to the conditional fragment probability), which should be applied at the fragment, rather than the equivalence class level. Developing an appropriate approximate factorization that accounts for our modified model (or extensions of it) should be feasible, but as the current computational costs remain quite reasonable, we leave that to future work.

**Figure 1.**
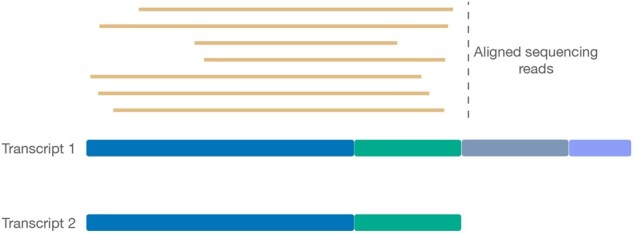
A toy example demonstrating how removing the length effect from the probabilistic model can increase fragment assignment ambiguity in situations where human intuition may be clear. In this case, all of the sequenced reads align equally well to Transcript 1 and Transcript 2 (since Transcript 2 is, in fact, a proper prefix of Transcript 1). Given the totality of coverage, it is clear that these reads likely derive from Transcript 2, as the likelihood of generating this many reads from Transcript 1, but never sequencing beyond the second exon, is quite low. The model, however, does not encode this intuition and does not convey a preference for Transcript 2 over Transcript 1 as an explanation for these reads.

**Figure 2.**
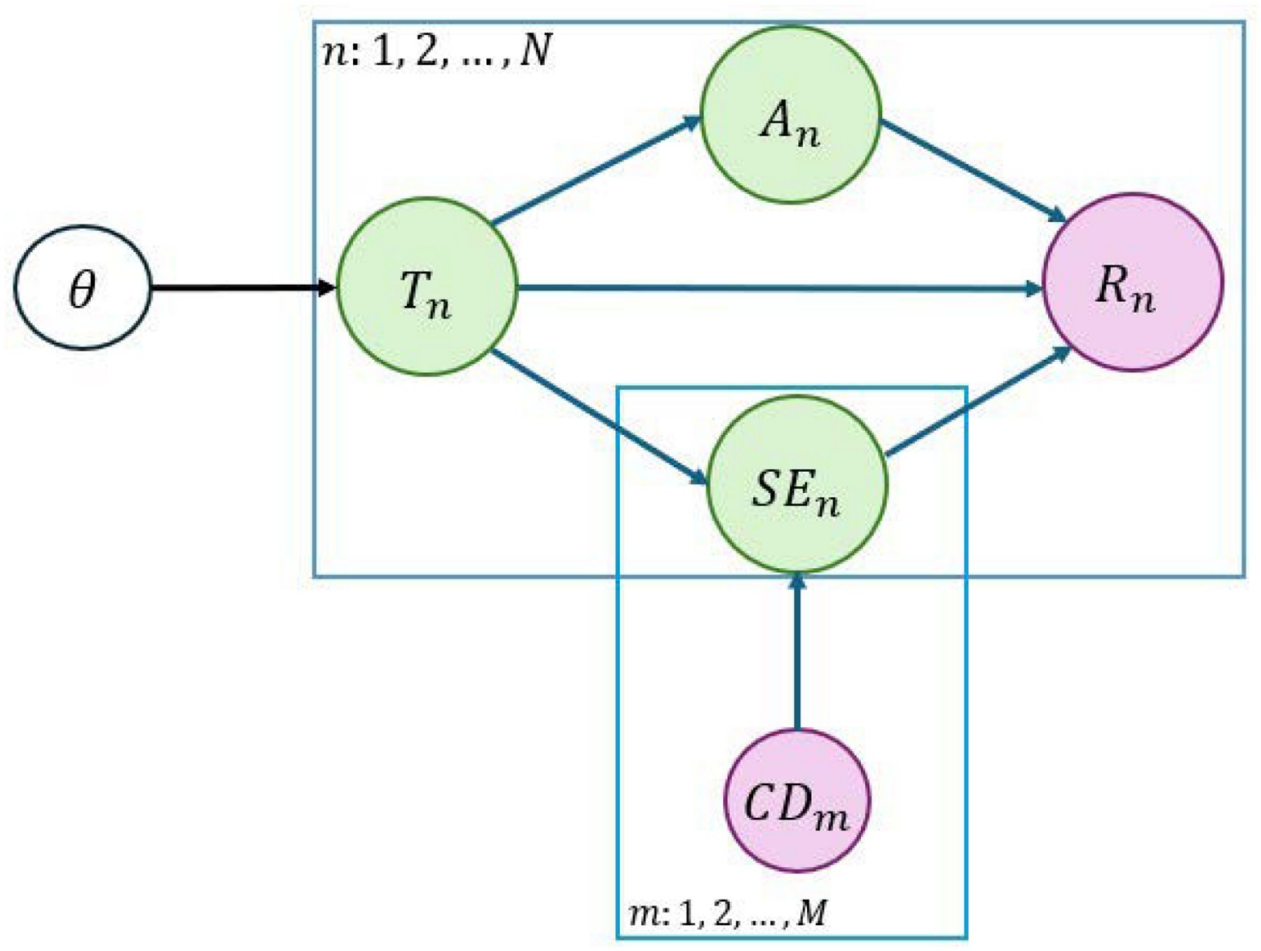
Graphical model for an improved generative model of RNA-seq long reads. θ represents the desired isoform expression level parameter. The latent variables $T_{n}$, $A_{n}$, and $SE_{n}$ correspond to the isoform, alignment score, and start-end positions, respectively. The observed variables $R_{n}$ and $CD_{m}$ are associated with the read and coverage model, where *m* and *n* stand for the number of isoforms and reads, respectively. Green circles represent latent variables, white circles denote desired parameters, and pink circles indicate observed variables.

The proposed generative model is illustrated in Fig. 2. The key insight behind our modification to the underlying model is that, in the short read sequencing model, the length dependence of the sequencing rate parameter provides useful information to help differentiate different potential origins of a read, not just in read generation, but also in read assignment. That is, when a read aligns equally well to multiple transcripts, the model may assign a higher probability to the shorter transcript if the longer transcripts matching the read contain more unaligned regions where reads could have occurred but did not. Another way of viewing this is that the length parameter essentially penalizes uncovered stretches of transcripts where alignments do not occur. However, when we move to the long read model and remove this length dependence in the conditional probability of fragment generation, this discriminative component of the model is lost. This can lead to counter-intuitive situations where the model fails to differentiate between potential mapping locations where a human observer may have a clear preference—see Fig. 1, an illustrative example of such a case. However, we do not want to include a length dependence in the long read quantification model, since, in general, we do not expect one given the underlying protocol and we expect that the length will not have a direct effect on the probability of sampling reads from a transcript—other than the effect that may arise from e.g. secondary and tertiary molecule structure and other biochemical preferences.

Thus, we propose to incorporate a new term into the model that directly accounts for the coverage signal of the underlying transcripts. This term works to penalize large deviations in coverage over the body of a transcript when assessing the conditional probability of fragment assignment. This provides extra information to differentiate between potential allocations like those in Fig. 1, and to prefer more “intuitive” explanations for the reads when they are available. We note that, even without the coverage term, the oarfish model is distinct from previous models, as it derives a different probability based on alignment score, and does not incorporate an effective length term.

A plate diagram of our model is provided in Fig. 2, where $R_{n}$ and $CD_{m}$ are the observed variables representing the long read sequences and potential coverage pattern for the $m^{th}$ isoform, respectively. $\theta =\left[\theta_{1},..,\theta_{M}\right]$ is the vector of isoform abundances that will be estimated via maximization of the associated likelihood. Additionally, $T_{n}$, $A_{n}$, and $SE_{n}$ are the latent random variables representing the isoform, alignment score, and start-end positions of alignments, respectively. The likelihood function for the modified generative model is given in Equation (2). Also, the explanation of the notations used in the generative model and likelihood function can be found in Table 1.


(2) $$\begin{array}{c} \mathrm{Pr}(T,A,CD,SE,R|\theta )=\\ \;\prod_{n=1}^{N}\sum_{j\in A(r_{n})}\mathrm{Pr}(T_{n}=j|\theta )\mathrm{Pr}(A_{n}=a_{nj}|T_{n}=j)\\ \times \mathrm{Pr}(SE_{n}=se_{nj}|T_{n}=j,CD_{j})\\ \times \mathrm{Pr}(R_{n}=r_{n}|T_{n}=j,A_{n}=a_{nj},CD_{j},SE_{n}=se_{nj}) \end{array}$$

**Table 1.** Summary of notation for the generative model.

Notation	Explanation
θ	Vector of isoform abundance parameters.
M	Number of isoforms.
N	Number of reads.
$T_{n}$	Isoform random variable for read n.
$A_{n}$	Alignment score random variable for read n.
$SE_{n}$	Start-end position random variable for read n.
$CD_{m}$	Coverage distribution model for isoform m.
$R_{n}$	Read random variable for read n.
$A(r_{n})$	Set of all isoforms to which read n aligns.

As shown in Fig. 2, the (latent) random variable $T_{n}$, denoting the transcript selected for read generation, only depends on the isoform expression parameters $\theta =\left[\theta_{1},..,\theta_{M}\right]$. In other words, if we know the expression level of all isoforms in the transcriptome, then the probability that we have selected the $j^{th}$ isoform for sequencing is equal to its expression level or relative abundance $\theta_{j}$, so that $\mathrm{Pr}(T_{n}=j|\theta )=\theta_{j}$.

With the model in hand, the first step in the process of long-read quantification is to align the reads to the target transcriptome to be quantified. For this task, oarfish provides two different options. Either, the user can provide a BAM file with reads aligned to the target transcriptome using minimap2 (Li 2018) (we require that reads are collated by query name, so either the default output order of minimap2 or a name-sorted BAM file will suffice), or they can provide the raw reads and reference sequence directly, and oarfish will use the minimap2-rs (Guhlin 2024) library to perform alignment itself. Of course, it is possible to modify oarfish to accept the input from other aligners, or even to make the use of spliced alignments of the reads directly to the genome rather than to the transcriptome, but these are practical details that do not fundamentally affect the model being considered here. While k-mer-based methods are common in short-read quantification, we use alignments against the transcriptome because long reads can span multiple exons and often exhibit complex sequencing errors and splicing patterns. Additionally, positional and alignment-based information is important for our probabilistic model, and it is not apparent how it may be easily or robustly derived using only k-mer-based notions of compatibility.

To obtain a reasonable model for $\mathrm{Pr}(A_{n}=a_{nj}|T_{n}=j)$, we use the alignment score computed by minimap2 and encoded in the AS tag, rather than the MAPQ value, since—as discussed in (Langmead 2017)—aligners often do not provide reliable estimates of mapping quality. AS also reflects the raw alignment characteristics directly, which aligns better with our probabilistic modeling. This alignment score is an integer value that reflects the quality of the alignment between read n and transcript j, and may be positive or negative depending on the alignment parameters and read quality. Specifically, we consider all alignments of the $n^{\text{th}}$ read and obtain the maximum alignment score among them. Let $j^{\prime }$ be the alignment of the $n^{\text{th}}$ read having maximum AS, which we denote as ${\text{AS}}_{n}^{\text{max}}$. We then consider this conditional probability as 1, and model the probability of other alignments for this read to decrease exponentially as a function of the alignment score. This falloff still assigns some probability to slightly suboptimal alignments and essentially discards highly suboptimal alignments. Specifically, the function used to obtain $\mathrm{Pr}(A_{n}=a_{nj}|T_{n}=j)$ is given in Equation (3).


(3) $$\mathrm{Pr}(A_{n}=a_{nj}|T_{n}=j)=\{\begin{array}{cc} \;\text{exp}\;\left(\frac{{\text{AS}}_{nj}-{\text{AS}}_{n}^{\text{max}}}{10}\right) & \forall j\in A(r_{n})\\ 0 & \text{otherwise}. \end{array}$$

In Equation (3), ${\text{AS}}_{nj}$ represents the alignment score of the $n^{th}$ read aligned to $j^{th}$ an isoform, and $A(r_{n})$ represents the set of all transcript indices to which read $r_{n}$ aligns.

One intuition behind our model is that, while we don’t expect an explicit length-based sampling effect, we would, all other things being equal, prefer uniform transcript coverage to highly non-uniform transcript coverage, if the corresponding read assignments explain the same set of reads. We observe that, in practice, the coverage pattern and uniformity vary substantially between the *potential alignments* of reads to different transcripts. For instance, Fig. 4, available as supplementary data at *Bioinformatics* online shows the coverage patterns of three transcripts ENST00000600659, ENST00000417088, and ENST00000449223 derived from aligning the Hct116 cell line dataset (see Results Section) to the transcriptome. The coverage patterns in Fig. 4, available as supplementary data at *Bioinformatics* online are extracted using IGV tools (Thorvaldsdóttir *et al.* 2013). Specifically, SAMtools (Danecek *et al.* 2021) was employed to acquire the depth of each position within the aforementioned three transcripts. The resulting data are depicted in Fig. 1, available as supplementary data at *Bioinformatics* online. It is evident, as observed in Figs 1 and 4, available as supplementary data at *Bioinformatics* online, that ENST00000417088 exhibits a coverage pattern closer to uniformity. In contrast, transcripts ENST00000600659 and ENST00000449223 display substantially less uniform patterns. Thus, it may be preferable to assign multimapping reads to ENST00000417088, at least when the alignment scores are close.

Overall, our approach will attempt to incorporate the coverage distribution itself into the allocation probability for each fragment. This idea itself is quite general, and we believe that it can be further explored to determine the best way to make use of the coverage profiles in read allocation. Here, however, we propose one specific and relatively simple model, and demonstrate that it helps to improve the quantification accuracy obtained by our approach.

We will assign to each alignment a conditional probability that is related to the coverage pattern along the transcript being considered. That is, when the coverage pattern is non-uniform, and we are considering the alignment of a read to a highly-covered section of the transcript that would increase that non-uniformity, we would like to *decrease* the conditional probability of this alignment (assuming that there are other locations compatible with the read, where allocating this read would contribute less to non-uniformity of coverage). Likewise, when we are considering the alignment of a read to a sparsely-covered section of the transcript, we may wish to *increase* the conditional probability of alignment to attempt to bring the coverage at the position covered by the read into closer concordance with the coverage of the rest of the transcript. The goal of such modifications to the conditional probabilities is to promote increased uniformity of coverage under the final allocations of reads. To accomplish this, first, we propose a model to compute a term inversely proportional to the coverage pattern for each transcript, which we elaborate below.

The process for acquiring the coverage distribution model for each transcript comprises the following steps:

**Align** the reads to the transcriptome.

**Segment** the transcript into disjoint intervals (default 100 bp).

**Count** the number of reads that partially or completely cover each segment. The count for each read present in a segment is computed as the fraction of the segment length that is covered by that read. Specifically, the count for the $i^{th}$ segment is given by $c_{i}=\sum_{k=1}^{N}\frac{\text{len}({\text{read}}_{k}\cap {\text{segment}}_{i})}{\text{len}({\text{segment}}_{i})}$, where *N*, ${\text{read}}_{k}$, and ${\text{segment}}_{i}$ represent the number of reads overlapping the $i^{th}$ segment, the $k^{th}$ read overlapping the $i^{th}$ segment, and the $i^{th}$ segment itself, respectively. To prevent numerically unstable probabilities, we add a small threshold equal to 1% of the total number of reads aligned to the transcript to each segment count.

**Compute the assignment probability for each segment**. The logistic function is used to compute the coverage probability based on the expected coverage across all segments. Specifically, if a segment coverage is larger than the expected value, we assign it a smaller probability, and conversely, if the segment coverage is smaller than the expected value, it receives a higher probability. To achieve this, we first calculate the expected coverage for a transcript as the average coverage across all segments. The expected coverage for the entire transcript is denoted as $E(c)=\frac{\sum_{i=1}^{K}c_{i}}{K}$, where *K* is the number of segments across the transcript and $c_{i}$ is the count of the $i^{th}$ segment. We compute $\delta_{i}=\frac{E(c)-c_{i}}{E(c)}$ as the input of the logistic function. The probability is then calculated as $p(\delta_{i},a)=\frac{1}{1+e^{-a\delta_{i}}}$, where $\delta_{i}$ is the input value calculated above and *a* is the growth rate of the function (a=2 by default).

An example visualization of these segment-level assignment probabilities is provided in Fig. 2, available as supplementary data at *Bioinformatics* online. As expected, the probability evaluated across each of the mentioned transcripts is roughly inversely proportional to the coverage density shown in Figs 1 and 4, available as supplementary data at *Bioinformatics* online.

Finally, with these coverage assignment probabilities in hand for each segment, we can compute the probability $\mathrm{Pr}(SE_{n}=se_{nj}|T_{n}=j)$ as the expected value of these probabilities across the relevant transcript. Here, $SE_{n}=(s_{n},e_{n})$ denotes the start and end positions of read n aligned to transcript j such that $0\leq s_{n}<e_{n}\leq L_{j}$, where $L_{j}$ is the length of transcript j. To compute $\mathrm{Pr}(SE_{n}=se_{nj}|T_{n}=j)$, we calculate the average of the probabilities over the segments from the start to the end of the alignment of the read on the transcript. The resulting probability is defined as $\mathrm{Pr}(SE_{n}=se_{nj}|T_{n}=j)=\sum_{i=b_{jn}}^{b_{jn}^{\prime }}w_{\mathrm{ijn}}\cdot P_{ij}\;\forall j\in A(r_{n})$, where $b_{jn}$ and $b_{jn}^{\prime }$ denote the start and end segments overlapping the alignment of read *n* on transcript *j*, $P_{ij}$ represents the coverage assignment probability for segment *i* along the length of transcript *j*, and $w_{\mathrm{ijn}}$ is the fraction of the *i*-th segment on transcript *j* overlapping read *n*.

Given this definition of the relevant probabilities, we apply the Expectation-Maximization (EM) algorithm (Dempster *et al.* 1977), as described inSupplementary Section E, to derive the maximum likelihood parameters corresponding to the transcript abundances.

## 4 Results

We conducted a detailed evaluation of our methodology using both simulated and experimental datasets. Our analysis includes a comparison against recent long-read-centric quantification methods, namely NanoCount (Gleeson *et al.* 2022), Bambu (Chen *et al.* 2023), lr-kallisto (Loving *et al.* 2025), TranSigner (Ji and Pertea 2024), IsoQuant (Prjibelski *et al.* 2023), and ESPRESSO (Gao *et al.* 2023). To assess the impact of the coverage model, we evaluate two variants of oarfish: oarfish (nocov), which excludes the coverage component and models only the transcript of origin and alignment score as latent variables; and oarfish (cov), which incorporates all latent variables shown in Fig. 2, including the transcript of origin, alignment score, and the start-end positions of alignments. To facilitate a rigorous comparison, we employ identical datasets and annotations as inputs for each methodology, and subsequently compute the correlation and error metrics for datasets derived from ONT and PacBio sequencing technologies. Additionally, we assess and compare the memory usage and runtime of each method.

### 4.1 Simulated dataset

To evaluate our approach against other methods in a situation where the ground truth is known, we generated simulated datasets targeting both the ONT and PacBio platforms. ONT datasets for 1DcDNA and direct-RNA sequencing were simulated using the NanoSim (Yang *et al.* 2017) tool, while PacBio datasets for RSII and SQ2-HiFi sequencing were generated with the TKSM (Karaoğlanoğlu *et al.* 2024) simulator. Both simulators use empirical short-read quantifications to seed the transcript abundance profiles for long-read simulations and incorporate error models derived from experimental data. For ONT, error models are based on H9 cell line data from the Singapore Nanopore Expression Project (Chen *et al.* 2021), while PacBio models use UHRR cell line data from the official PacBio repository (Pacific Biosciences 2024).

For ONT simulations, we restricted simulated reads to derive from protein-coding and long non-coding transcripts, but quantified abundance using the full transcriptome annotation. Additionally, we simulated an ONT dataset for the NA12878 sample (Workman *et al.* 2019) with both 1DcDNA and direct RNA protocols, similarly to what was done in the TranSigner manuscript (Ji and Pertea 2024). Further details on the simulation protocol are provided in Section C of the Supplementary.

Here, we present results for datasets seeded with the H9 and UHRR cell lines, simulating direct RNA (for ONT) and SQ2-HiFi (for PacBio) technologies, respectively. Results for the H9 1DcDNA and UHRR RSII datasets and the NA12878 1DcDNA and direct RNA datasets are provided in the supplement. All datasets were aligned to both the genome and transcriptome (O’Leary *et al.* 2016), as explained in Section F of the Supplementary. Our analysis includes correlation and error metrics, as well as comparisons of memory usage and runtime across methods.

#### 4.1.1 Correlation and error metrics analysis

To evaluate the performance of different methods, we assessed the strength and direction of linear and non-linear relationships between estimated and true isoform expression levels using the Pearson Correlation Coefficient and the Concordance Correlation Coefficient (CCC) to evaluate linear correlation and Spearman and Kendall correlation coefficients to capture rank-based relationships.

To evaluate the discrepancies between the quantification results of different methods and the ground truth, we calculated three error metrics: Root Mean Squared Error (RMSE), mean-Normalized Root Mean Squared Error (NRMSE), and Mean Absolute Relative Difference (MARD). Table 2 presents the correlation and error metrics for each quantification method applied to direct-RNA and SQ2-HiFi reads, while Table 1, available as supplementary data at *Bioinformatics* online shows the results for 1DcDNA and RSII datasets. For each metric, the highest correlation values and lowest error values are highlighted in bold. Across all metrics, oarfish, both with and without coverage modeling (i.e., applying our coverage-based conditional assignment probability), consistently outperform existing methods.

**Table 2.** Evaluation metrics on the direct RNA (ONT) and SQ2 HiFi (PacBio) simulation reads obtained from H9 and UHRR cell lines for the evaluated methods.

Method	Spearman ρ		Pearson(log(1+x))		CCC		Kendall-τ		RMSE		NRMSE		MARD	
	dRNA	HiFi	dRNA	HiFi	dRNA	HiFi	dRNA	HiFi	dRNA	HiFi	dRNA	HiFi	dRNA	HiFi
oarfish (cov)	**0.93**	**0.95**	**0.98**	**0.98**	**0.98**	**0.98**	**0.92**	0.91	**89.02**	**55.38**	**1.32**	**0.70**	**0.03**	**0.09**
oarfish (nocov)	0.91	**0.95**	**0.98**	**0.98**	**0.98**	**0.98**	0.90	**0.92**	187.61	132.34	2.78	1.68	0.04	**0.09**
NanoCount	0.55	0.62	0.73	0.73	0.70	0.69	0.49	0.51	905.12	811.30	13.42	10.30	0.32	0.58
NanoCount (nofilt)	0.58	0.81	0.83	0.90	0.82	0.90	0.52	0.70	355.99	248.79	5.28	3.16	0.33	0.45
bambu	0.82	0.92	0.92	0.95	0.92	0.95	0.79	0.87	321.73	150.09	4.77	1.91	0.08	0.12
lr-kallisto	0.70	0.88	0.90	0.92	0.90	0.92	0.67	0.82	478.95	455.35	7.10	5.78	0.14	0.17
TranSigner	0.56	0.80	0.80	0.91	0.77	0.90	0.50	0.69	260.78	129.35	3.87	1.64	0.43	0.46
IsoQuant	0.89	0.91	0.95	0.92	0.94	0.92	0.88	0.85	360.78	324.68	5.35	4.12	0.05	0.16
ESPRESSO	0.84	0.84	0.89	0.88	0.84	0.82	0.81	0.78	949.21	473.92	14.08	6.02	0.10	0.24

Note: The highest correlations and lowest errors are shown in bold in each column.

The performance differences are especially noticeable in the ONT direct-RNA dataset. Given the higher error rates of ONT technology, these results indicate that while oarfish is a top-performing method on ultra-high quality data, it also yields substantially better estimates, particularly for datasets with higher sequencing error rates. For more accurate datasets like SQ2-HiFi reads, oarfish without coverage modeling shows a modest (approximately 1%) improvement in correlation values compared to the version with coverage modeling. Similarly, in the 1DcDNA dataset, which has a lower error rate than RSII, oarfish without coverage modeling provides a minor advantage (around 1%) in linear correlation values.

The evaluated metrics may co-vary in complex ways. Thus, we also produced 2D plots, plotting the Spearman correlation in relation to various other metrics, including Pearson correlation, CCC, Kendall, 1/NRMSE, and 1/MARD, to see how methods perform under two different metrics simultaneously. These plots are designed such that methods closer to the top-right corner of the figures exhibit better performance. As illustrated in Figs 5 and 6, available as supplementary data at *Bioinformatics* online, oarfish with coverage modeling consistently achieves top performance across ONT (both direct RNA and 1D cDNA) data from the H9 cell line and PacBio (both SQ2-HiFi and RSII) sequencing datasets from the UHRR cell line sample. Similar results are also observed for 1DcDNA and direct RNA simulations seeded from the NA12878 sample (Table 2 and Fig. 7, available as supplementary data at *Bioinformatics* online).

Figures 8–10, available as supplementary data at *Bioinformatics* online show density plots, accompanied by best-fit regression lines and the calculated Pearson correlation coefficients for both ONT (both direct RNA and 1D cDNA) and PacBio (both SQ2-HiFi and RSII) simulations seeded from the H9 and UHRR cell line samples. In these plots, the simulated counts are displayed on the x-axis and the estimated counts are displayed on the y-axis, both on the log  scale. While all methods tend to show concentration around the diagonal (and the best-fit line often closely matches the diagonal), we observe that the oarfish variants tend to display the highest correlations and tightest density about the diagonal.

Finally, we evaluate the effectiveness of each method in identifying expressed transcripts across datasets by computing precision-recall (PR) curves, where expressed transcripts are categorized as positive instances, and unexpressed transcripts are classified as negative instances. Here, the “score” assigned to each transcript under each method is simply the estimated abundance. We plot these curves and compute the Area Under the Curve (AUC) and average precision (AP) values for each method. The AUC values indicate each method’s ability to differentiate between expressed and unexpressed transcripts, while the AP values provide a summary of overall performance by capturing precision at various recall levels.

Figure 11, available as supplementary data at *Bioinformatics* online shows the precision-recall curves for the ONT and PacBio sequenced datasets from the H9 and UHRR cell lines, while Fig. 12, available as supplementary data at *Bioinformatics* online presents the results for the ONT sequenced datasets seeded from the NA12878 samples. The results demonstrate that oarfish, both with and without coverage modeling, consistently outperform other methods, achieving higher AUC scores and superior average precision across all datasets.

#### 4.1.2 Runtime and memory usage

**Figure 3.**
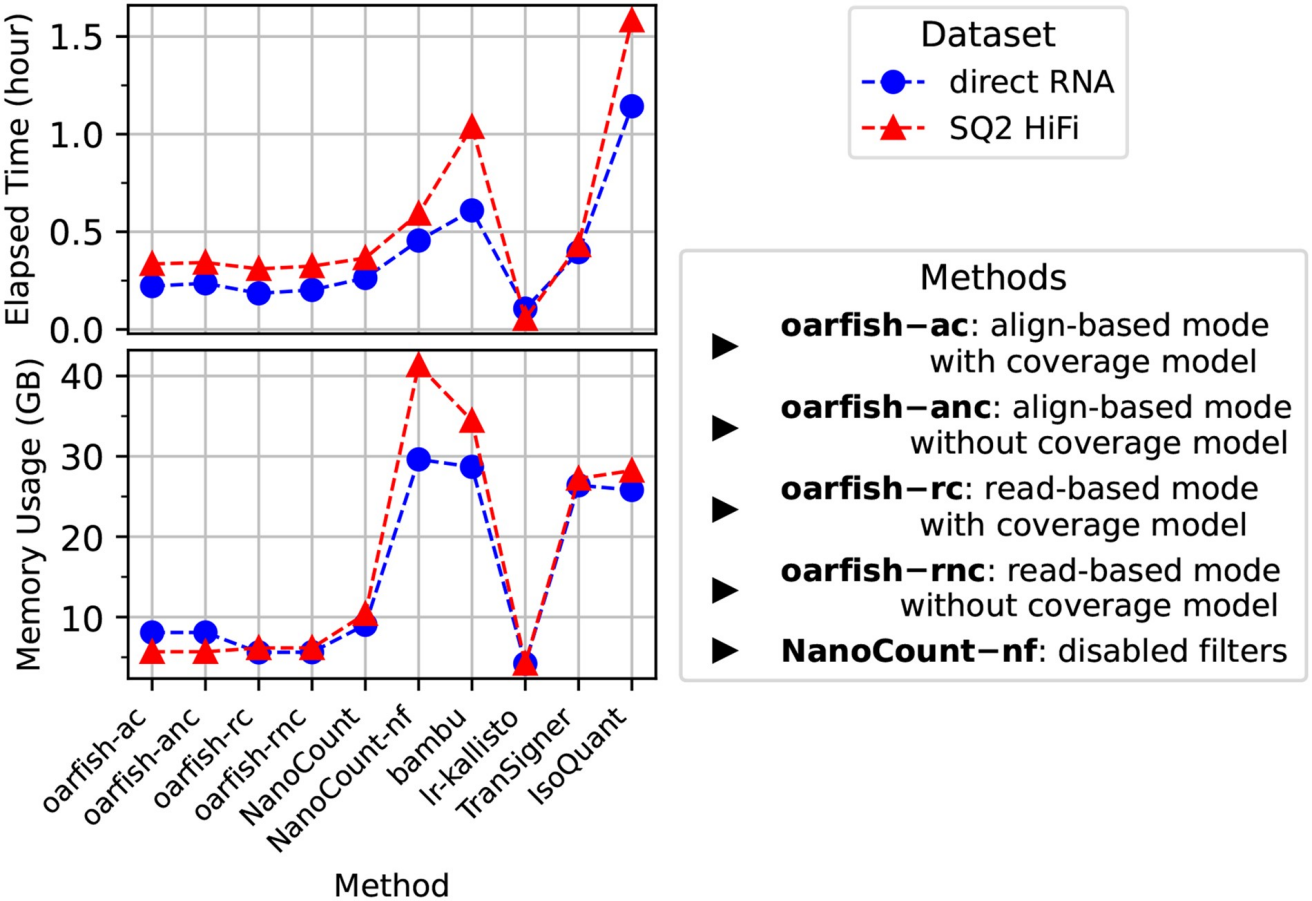
Performance metrics (elapsed time and peak memory usage) for the proposed and alternative methods using the direct-RNA and SQ2-HiFi simulated datasets from H9.

We evaluated the time and memory requirements of the methods across the simulated ONT and PacBio datasets. Figure 3 and Fig. 13, available as supplementary data at *Bioinformatics* online present these benchmarks for the direct-RNA and SQ2-HiFi datasets, as well as the 1DcDNA and RSII datasets, all simulated from the H9 and UHRR cell lines dataset. Additionally, Fig. 14, available as supplementary data at *Bioinformatics* online shows the results for the 1DcDNA and direct-RNA simulations seeded from the NA12878 sample.

There are substantial differences in time and memory usage among the tools. Specifically, ESPRESSO exhibited the highest resource demands, with runtimes approaching 14–30 hours and memory consumption nearing 140–160 GB. Yet, it makes little sense to directly compare the resource requirements of ESPRESSO with the other tools considered here, as ESPRESSO performs both transcript quantification *and* assembly, and, unlike bambu, the assembly module cannot be disabled. Figures 3, Figs 13 and 14, available as supplementary data at *Bioinformatics* online provide a comparison among quantification-only tools. Amongst the tested methods, lr-kallisto, which relies on pseudoalignment rather than full nucleotide-level alignment, required the least time and memory to complete quantification, as expected (generally 4–11 minutes and ∼4GB of RAM). Oarfish also demonstrated very fast quantification and low memory requirements, completing the analyses in just 6–22 minutes while using only ∼5 GB of memory across datasets. Specifically, we find that when oarfish is provided with the raw sequencing reads and reference, and allowed to perform the alignment itself using minimap2-rs, it exhibits lower runtime and memory usage than is required to first align with minimap2 and then quantify from the produced BAM file. This is likely due to the ability to avoid the creation, writing, and reading of the intermediate BAM file, as well as the ability to avoid the largely serial process of reading the BAM file to populate internal data structures. The remaining methods were slower (some substantially) and required more (some substantially) memory than oarfish and lr-kallisto.

### 4.2 Experimental dataset

We evaluate oarfish (and the other methods) across three diverse experimental datasets: Hct116, UHRR, and SH-SY5Y, each derived from distinct sources. The Hct116 data come from the Singapore Nanopore Expression Project (Chen *et al.* 2021), publicly-available sequencing samples using the Kinnex full-length RNA kit (Pacific Biosciences 2024) provides long reads for the UHRR sample, and Wang *et al.* sequence SH-SY5Y using their novel TEQUILA-seq (Wang *et al.* 2023) protocol. The Hct116 cell line dataset (Chen *et al.* 2021) and the UHRR dataset (Pacific Biosciences 2024) are sequenced using ONT and PacBio technologies, respectively, allowing us to present results across both platforms. Meanwhile, the SH-SY5Y cell line dataset (Wang *et al.* 2023) incorporates multiple ONT sequencing technologies, including TEQUILA-seq, a new targeted sequencing method. Details on dataset selection and alignment methods are provided inSupplementary Section D and Section F, respectively. Our analysis covers correlation and error metrics, as well as a comparative assessment of memory usage and runtime across methods.

#### 4.2.1 Correlation and error metrics analysis

Short-read RNA-seq is a well-established technology that provides high sensitivity in quantifying transcript and gene expression due to its deep sequencing coverage across transcripts. This sensitivity and the established nature of short-read technologies make them a reasonable point of comparison for evaluating long-read sequencing and quantification approaches. However, short-read sequencing and quantification are not uniformly reliable for all transcripts across the transcriptome. To stratify transcripts with respect to the reliability of their short-read quantification estimates, we use the inferential relative variance (InfRV) metric introduced by Zhu *et al.* (2019).

InfRV quantifies the relative uncertainty in transcript abundance estimates by measuring the variation of inferred counts or expression levels across inferential replicates (Gibbs samples in this case). InfRV is defined as $\text{InfRV}=\max(\sigma^{2}-\mu ,0)/\mu$, where $\sigma^{2}$ and μ represent the sample variance and mean across the Gibbs samples, respectively. To ensure stability in and guarantee positivity for log-transformation, as recommended in Zhu *et al.* (2019), we add a pseudocount of 5 to the denominator and adjust all InfRV values by 0.01 so that we obtain $\text{InfRV}=(\max(\sigma^{2}-\mu ,\;0)/(\mu +5))+0.01$

In practice, a high InfRV value indicates greater uncertainty in quantification, while a low InfRV suggests more stable and reliable abundance estimates. We use InfRV values from short-read quantification to identify the most reliably quantified transcripts, providing a robust point of comparison for evaluating long-read quantification accuracy.

We use the quartile values of the full InfRV distribution—the minimum, 25th percentile, median, 75th percentile, and maximum—to stratify transcripts by levels of uncertainty in the short-read data. This creates five subsets with progressively higher uncertainty. In most datasets assessed, the minimum and 25th percentile values are equivalent, which omits the (minimum, 25^th^ percentile) subset from the plots. Consequently, each subset represents a stepwise increase in transcript uncertainty, allowing for a nuanced evaluation of reliability across InfRV thresholds.

To evaluate the long-read experimental dataset against short-read quantification results, we compute the Spearman and Kendall correlations, the Pearson and CCC correlations, and the RMSE, NRMSE, and MARD metrics. For simplicity, we present only the Spearman correlation, Pearson correlation, and MARD here, with the remaining metrics available inSupplementary Section H.

**Figure 4.**
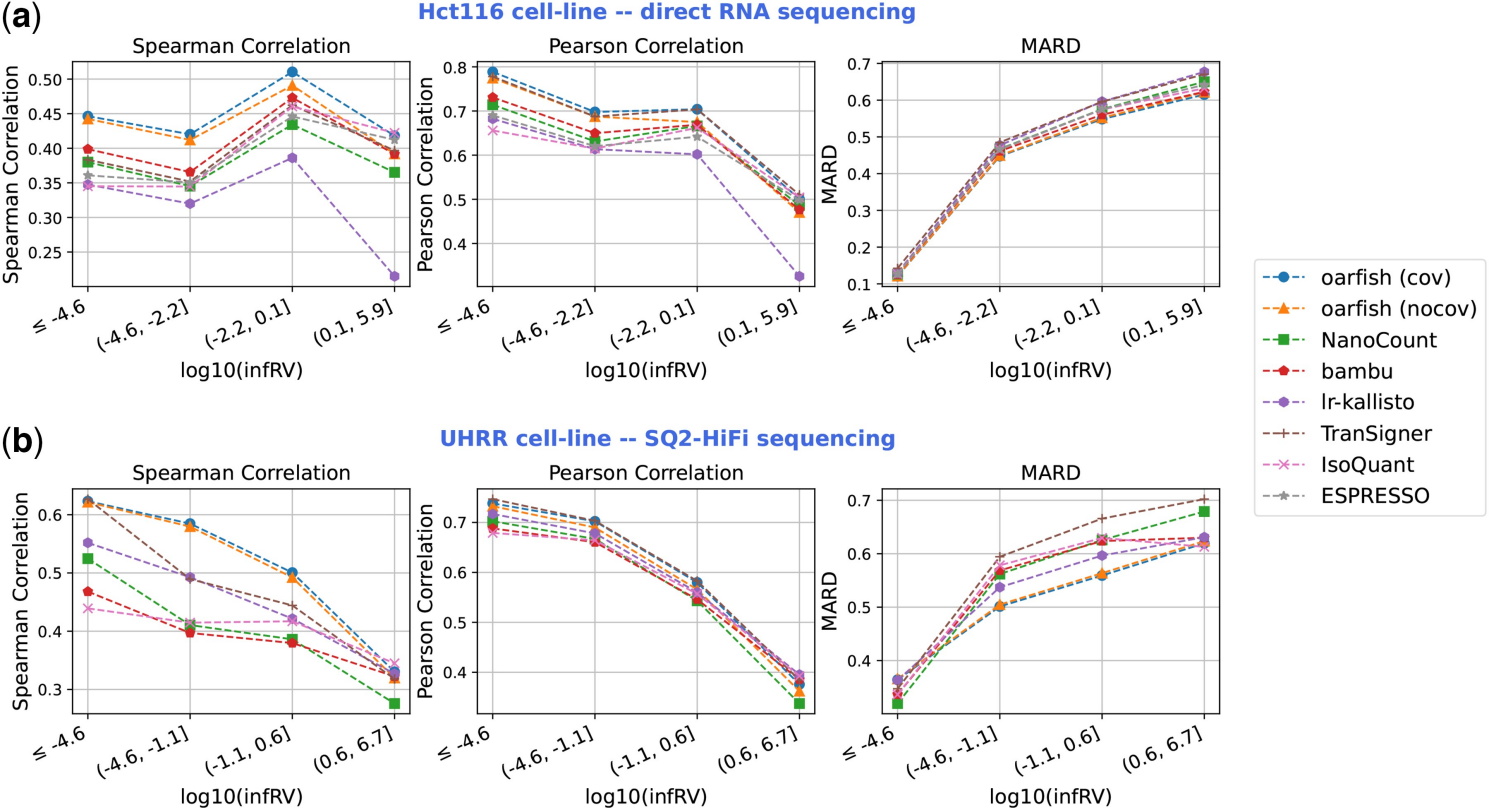
Spearman correlation, Pearson correlation, and MARD between long-read and short-read quantification results on different subsets of InfRV values on all the transcripts. (a) The correlation and error metrics on the ONT long-read RNA-seq dataset sequenced with the direct RNA protocol from the Hct116 cell line. (b) The correlation and error metrics on the PacBio long-read RNA-seq dataset sequenced with the SQ2-HiFi protocol from the UHRR dataset.

To assess the methods on both ONT and PacBio sequencing data, we used the direct RNA and direct cDNA ONT sequencing data obtained from the Hct116 cell line (Chen *et al.* 2021) and the Kinnex full-length RNA kit dataset for Universal Human Reference RNA (Pacific Biosciences 2024) (both SQ2-HiFi and Revio-HiFi), respectively. As shown in Fig. 4 and Fig. 16, available as supplementary data at *Bioinformatics* online, oarfish with the coverage model achieves the highest Spearman and Pearson correlations and the lowest MARD values compared to other methods, especially for transcripts with less short-read quantification uncertainty. Furthermore, Fig. 17 and 18, available as supplementary data at *Bioinformatics* online show that oarfish with the coverage model also outperforms other methods on additional metrics, including Kendall correlation, CCC, RMSE, and NRMSE. TranSigner demonstrates the best Pearson and CCC correlations on SQ2-HiFi/Revio-HiFi reads, though those results are only marginally higher (∼1%) than oarfish. Overall, these results indicate that oarfish with the coverage model is a top-performing method, consistently on par with or outperforming the other methods across all seven metrics.

Furthermore, Chen *et al.* (2021) identified transcripts with the highest average expression levels among those associated with a gene in either long-read or short-read data, which they define as *major transcripts*. For further analysis, we evaluated the correlation and error metrics specifically for major transcripts across different InfRV value subsets in both direct cDNA and direct RNA datasets from the Hct116 cell line. As shown in Fig. 19 and 20, available as supplementary data at *Bioinformatics* online, oarfish with the coverage model consistently outperforms other methods across both linear and non-linear correlation and error metrics, particularly for major transcripts with the lowest InfRV values.

The Hct116 sample further includes sequin spike-in transcripts with known concentrations, which also serve as benchmarks for evaluation. Table 3, available as supplementary data at *Bioinformatics* online indicates that all methods perform comparably, presumably due to the limited set and relative ease of quantification of the 164 sequins. Figure 15, available as supplementary data at *Bioinformatics* online highlights the oarfish’s superior performance in paired metric comparisons. We observe similar results when evaluating density plots (Fig. 21, available as supplementary data at *Bioinformatics* online) and precision-recall curves (Fig. 22, available as supplementary data at *Bioinformatics* online) in assessing different methods’ quantification of sequin transcripts.

We also assess the methods on the SH-SY5Y cell line dataset, which includes various ONT sequencing protocols, including the novel targeted sequencing protocol TEQUILA-seq (Wang *et al.* 2023). These results are available in theSupplementary Section H.5.

We observe similar runtime & memory usage results in the experimental data as in the simulated data, with further details described in theSupplementary Section H.6.

## 5 Conclusion

In this paper, we have introduced an enhanced model and tool for transcript quantification from long-read RNA-seq data. Because of differences in the fundamental generative process (i.e. the absence of systematic fragmentation in long-read protocols and hence the lack of a length dependence in the abundance parameters), existing short-read quantification tools—at least those that have not been explicitly retrofitted for long-read quantification—are not well-suited to the task. At the same time, while numerous long-read transcriptome analysis methods have been developed, most of these tools have prioritized identification over quantification. Yet, accurate quantification from long reads remains an unresolved challenge, and our emphasis here has been on improving the quantification model itself, recognizing its significance alongside identification.

While existing long-read quantification tools augment short-read quantification models with various additions, none of these tools incorporates a model that explicitly accounts for the distribution of read coverage for individual transcripts. As we demonstrate, the read coverage pattern is a crucial piece of evidence that can shed light on the likelihood of sequenced reads originating from specific regions of transcripts. The results demonstrate that oarfish, with the coverage distribution model, delivers a substantial improvement in precision and accuracy over existing methods, particularly as far as can be assessed via simulations and also for datasets with higher error rates. Additionally, oarfish demonstrates the fastest execution speed and lowest memory usage amongst the alignment-based tools, not far from that of the only pseudoalignment-based tool tested. We observed that the correlation metrics for oarfish with and without the coverage model tend to be somewhat similar, especially with the highest fidelity data. However, the error metrics—such as RMSE and NRMSE—are nearly halved when applying the coverage model. Additionally, we found that the benefits of coverage modeling are more evident in datasets with lower overall fidelity and higher alignment ambiguity, such as ONT direct RNA, compared to higher fidelity datasets like PacBio HiFi. An important direction for future investigation is analyzing how the number of aligned reads impacts the effectiveness of the coverage model. We hypothesize that with increased read depth, the coverage model becomes more informative and may also yield improved correlation metrics compared to oarfish without coverage modeling.

Although our proposed model improves quantification accuracy, it is just a first step, with several promising directions for future work. Theoretically, our coverage model is simple—based on mapping deviation from expected coverage to a probability using a logistic function, and *static* during inference, as it does not update after the initial alignments. However, as inference progresses, we gain information about the likely origin of multimapping reads. A valuable extension would be to dynamically update coverage profiles during inference based on the inferred read allocations. This approach, though challenging in terms of computational efficiency and convergence, has been successfully applied in learning fragment length distributions (Li and Dewey 2011) and bias models (Roberts and Pachter 2013, Patro *et al.* 2017) for short-read quantification and could be promising for coverage distributions as well.

Also, as we have mentioned earlier, oarfish considers an alignment-level model for the purposes of optimization—that is, there is no summarization or aggregation of alignments into, e.g. equivalence-classes (Turro *et al.* 2011, Patro *et al.* 2014, Bray *et al.* 2016, Patro et al. 2017, Zakeri *et al.* 2017). As demonstrated by the time and memory efficiency of oarfish, this does not seem to pose a practical impediment to efficient implementation. Nonetheless, while existing standard factorizations of the likelihood would interfere with our coverage modeling, an interesting practical direction for future work is designing a factorization that is appropriate for application under our coverage model (or a *dynamic* variant of it). Such an approach could even further improve the computational efficiency of oarfish.

## Supplementary Material

btaf240_Supplementary_Data

## Data Availability

All code used to reproduce the experiments and generate the results is available at: https://github.com/COMBINE-lab/oarfish-paper-scripts. The simulated dataset used in this study is available at: https://doi.org/10.5281/zenodo.15099250, https://doi.org/10.5281/zenodo.15099278, https://doi.org/10.5281/zenodo.15099283. The experimental dataset used in this study is available from: The Singapore Nanopore Expression Project, CBI SRA database under accession number SRR950078, GEO under accession number GSE213984. More information can be found in the supplementary file.
