# Supplementary material for: Oarfish: enhanced probabilistic modeling leads to improved accuracy in long read transcriptome quantification
Source: Bioinformatics. 2025 Jul 15;41(Suppl 1):i304–13. doi: 10.1093/bioinformatics/btaf240 (PMC12261437; doi:10.1093/bioinformatics/btaf240)
Supplement: btaf240_Supplementary_Data [file btaf240_supplementary_data.pdf]

Supplementary material for `Oarfish`: Enhanced probabilistic modeling leads to improved accuracy in long read transcriptome quantification

A Software versions for simulation, alignment and quantification tools

- `NanoSim`: `v3.1.0`
- `TKSM`: `v0.6.1`
- `Badread`: `v0.4.1`
- `salmon`: `v1.9.0`
- `minimap2`: `2.28-r1209`
- `oarfish`: `5f9724627b22558322b80ca6e0be087597b3043b`
- `NanoCount`: `v1.1.0`[1]
- `bambu`: `v3.4.1` (in `R` v4.4.1)
- `kallisto`: `v0.51.1`
- `bustools`: `v0.43.2`
- `TranSigner`: `v1.1.0`
- `IsoQuant`: `v3.4.1`
- `ESPRESSO`: `v1.5.0`

B Repository for reproducing the experiments

All code used to generate the results for each dataset (via the Snakemake workflow), perform the analysis and generate the paper figures (via Jupyter notebooks), and simulate datasets (using scripts run with `Task`) is available at: `https://github.com/COMBINE-lab/oarfish-paper-scripts`. Besides that, the simulated dataset used in this paper is accessible at: `https://doi.org/10.5281/zenodo.15099250`, `https://doi.org/10.5281/zenodo.15099278`, and `https://doi.org/10.5281/zenodo.15099283`.

C Simulated Dataset

We used the `nanosim` simulator [52] to generate ONT datasets for both 1D-cDNA and direct-RNA sequencing protocols, using the H9 cell line from the Singapore Nanopore Expression Project [9] and NA12878 sample from the nanopore wgs consortium [50]. Additionally, the `TKSM` simulator [21] was used to simulate PacBio datasets for the RSII and SQ2-HiFi sequencing protocols, based on the UHRR cell line [9]. The procedures for each simulation are outlined below.

C.1 ONT Simulation Steps

The `nanosim` simulation processes involve the following steps:

- **Download Experimental Data:** We obtained ONT 1D-cDNA and direct-RNA sequencing reads from H9 cell line via the Singapore Nanopore Expression Project [9], and from NA12878 sample, as reported by Workman et al. [50]. These datasets were used as experimental data for training the simulator.
- **Download Reference Genome & Transcriptome:** We downloaded the GRCh38 (RefSeq 110) human reference genome and transcriptome [35]. The transcriptome was filtered to include only protein-coding and long non-coding RNA (lncRNA) transcripts, ensuring that only relevant sequences were used in the simulations.
- **Alignment & Quantification:** The ONT datasets were aligned to the reference transcriptome using minimap2 [26], and transcript abundances were quantified with salmon [37]. These quantifications were used to generate transcript-level abundance estimates for input into the simulation.
- **Training the Simulation Model:** Using the experimental ONT read dataset, we trained the `nanosim` model to capture key sequencing characteristics such as read length distribution, error profiles, and other relevant biases. This training process was essential for generating realistic synthetic reads.
- **Simulation of ONT datasets:** Finally, we simulated the ONT reads using the trained `nanosim` model. The simulation generated synthetic reads that reflected the characteristics of the ONT 1D-cDNA and direct-RNA sequencing protocols, ensuring a realistic replication of the original dataset.

C.2 PacBio Simulation Steps

The `TKSM` simulation processes involve the following steps:

- **Download Experimental Data & Reference Genome/Transcriptome:** We obtained PacBio RSII and SQ2-HiFi sequencing reads from UHRR sample via the official PacBio website [36]. These datasets were used as experimental data for training the simulator. Besides that, We used the GRCh38 (RefSeq 110) human reference genome and transcriptome [35] in our simulation process.
- **Alignment & PAF File Generation:** The PacBio datasets were aligned to the reference transcriptome using minimap2 [26], and then convert the SAM files to PAF format for downstream processing.
- **Transcript Quantification:** Transcript abundance is estimated from the alignment files using `tksm abundance`, producing quantification results for each sample.
- **Transcriptome Simulation:** Molecules are transcribed from the reference GTF file using the quantified transcript abundances. The transcription process generates simulated molecules with a specified number of molecules per sample.

[1] Version 1.1.0 was not available via pip or bioconda at the time we conducted benchmarking, so it was installed from source using the head of the “master” branch, corresponding to git commit hash `3ae643c6aae87166b56269166c6c8e472b9ec0e7`.

- **Error, Qscore, & Truncation Models:** Using the experimental PacBio read dataset, we trained the `Badread` model to capture key sequencing characteristics such as read length distribution, error profiles, and other relevant biases. This training process was essential to accurately simulate sequencing errors and quality scores for generating realistic synthetic reads. Besides that, we used `tksm model-truncation` to construct a KDE-based truncation model from the PAF alignment files.
- **PolyA Tail Addition, Truncation & Shuffling:** PolyA tails are added to the simulated molecules based on the specific sequencing platform (RSII or SQ2-HiFi) using `tksm polyA`. Also, The molecules are shuffled and truncated using `tksm shuffle` and `tksm truncate`, applying the previously generated KDE-based truncation model.
- **Sequencing Simulation:** Finally, sequencing reads are simulated using `tksm sequence`, incorporating the appropriate error and qscore models for RSII and SQ2-HiFi datasets.

D Experimental Dataset

The experimental datasets on which we evaluated `oarfish` and the other tools — Hct116, UHRR, and SH-SY5Y — were selected for the following reasons:

The Singapore Nanopore Expression Project[9]: We selected the dataset as it has been designed, collected, and distributed as a community resource meant to be particularly useful for aiding in method development and benchmarking. Additionally, the corresponding paper provides a useful classification with regards to the major isoforms of each gene within each cell line / tissue, which can be used to "zoom in" on quantification estimates, and highlight differences in performance that may be otherwise hidden or obscured in transcriptome-wide assessments. This determination of major isoforms relies on the average expression levels for all isoforms associated with a gene, utilizing either long read or short read data. This specificity enables the assessment of most active isoforms individually, in addition to the evaluation of all isoforms collectively within each sample. Furthermore, synthetic spike-in sequin data with specific concentrations has been incorporated into certain datasets, including Hct116 (Colon cell line), K562 (Leukocytes cell line), and MCF7 (Breast cell line). This inclusion facilitates the assessment of isoform expression level quantification within an experimental dataset using external controls with known molar concentrations. In the analyses herein, we used both short read and long read RNA-seq datasets obtained from the Colon cell line, denoted as Hct116.

Kinnex full-length RNA kit for isoform sequencing [36]: We selected data from this study for its inclusion of long-read RNA-seq data generated using the latest PacBio sequencing protocol, which offers higher base-calling accuracy (i.e., lower error rates) than data from Oxford Nanopore Technology (ONT), while potentially exhibiting distinct artifacts or biases. By combining PacBio long-read data with short-read RNA-seq data, we establish a more robust framework for evaluating our proposed method across both PacBio and ONT technologies. The Kinnex full-length RNA kit, used in this recent PacBio sequencing protocol, addresses the previous limitation of low throughput in PacBio datasets, making them more comparable to ONT data. For our analysis, we utilized datasets from Universal Human Reference RNA (UHRR) sample, sequenced using both RSII and Sequel II-HiFi platforms. UHRR is a fixed-molar mixture of RNA extracted from various human cell lines, providing a consistent and representative sample for benchmarking. Although the short-read UHRR data was not available on the same platform as the PacBio data, we obtained it from the NCBI SRA database under accession number SRR950078.

TEQUILA-seq[46]: TEQUILA-seq is a low-cost sequencing method implemented on the ONT platform which improves isoform coverage and quantification in the context of *targeted sequencing* by mitigating the low throughput constraint of current long-read sequencing platforms. Consequently, datasets obtained from [46] serve as a valuable resource for demonstrating the applicability of our method, given its reliance on isoform coverage distribution. The improved precision in coverage distribution is important for enhancing the accuracy of results obtained from the proposed method. Additionally, the inclusion of synthetic spike-in SIRV data with known concentrations further augments the dataset's utility. This study exclusively utilizes samples derived from human neuroblastoma cell lines (SH-SY5Y), including both short read RNA-seq data and long read RNA-seq data obtained through three distinct sequencing methods: direct-RNA, 1D cDNA, and TEQUILA-seq; all implemented on the ONT platform.

E EM algorithm

The EM algorithm used in `oarfish` initializes transcript abundances uniformly across all transcripts by dividing the total number of aligned reads by the number of transcripts. The EM iterations proceed until convergence is achieved, either when the maximum relative change in the abundance estimate of any transcript falls below a threshold of 10^{-3}, or after a maximum of 1000 iterations. Both of these values are default values in `oarfish`, and can be adjusted via the `convergence_thresh` and `max_em_iter` command-line arguments, respectively. Unlike RSEM, `oarfish` does not incorporate fragment length distributions or quality score modeling, but does include an alignment score model that necessarily tolerates insertions and deletions in the alignment. A high-level description of the EM can be given as follows:

1. Initialization: initialize the isoforms' relative abundances $\theta = [\theta_0, \theta_1, \ldots, \theta_M]$ (different initializations are possible, but we used the uniform initialization in oarfish such that each transcript is initialized with a value equal to the total number of alignments divided by the number of transcripts.)
2. E-step: For each read r_n let $\mathcal{A}(r_n)$ be the set of transcripts to which it aligns. We compute the posterior probability that the read originated from transcript $j \in \mathcal{A}(r_n)$ based on the current abundance estimate $\theta^{(t)}$ as follows: $\omega_{nj}^{t} = \frac{Pr(r_n|t_j) \times \theta_j^{(t)}}{\sum_{k \in \mathcal{A}(r_n)} Pr(r_n|t_k) \times \theta_k^{(t)}}$. Here, $Pr(r_n|t_j)$ is the likelihood of observing read r_n given transcript t_j and is computed as the production of the normalized alignment score and optional coverage probability. These posterior probabilities ω_{nj}^{t} represent the soft assignments which is the expected fractional contribution of read r_n to transcript t_j under the current model parameters.
3. M-step: Using the expected assignment weights from E-step, we update the abundance estimate for each transcript by summing the assignments across all reads as follows: $\theta_j^{(t+1)} = \sum_{n=1}^{N} \omega_{nj}^{(t)}$. Here, the $\theta_j^{(t+1)}$ is the updated abundance of transcript t_j at iteration $t+1$. The updated abundances are then used in the next E-step.
4. Convergence: The algorithm alternates iteratively between E-step and M-step until the difference between $\theta^{(t)}$ and $\theta^{(t+1)}$ is less than a specific threshold (default $= 10^{-3}$), or the number of the iterations exceed some particular value (default $= 1000$).

F Alignment and Quantification Methods

F.1 Alignment Code

In this manuscript, we benchmarked our proposed method using both simulated and experimental datasets, each comprising both ONT and PacBio sequencing data. The details of the simulated dataset generation are provided in Section C of the appendices. For the experimental datasets, we used data from three distinct cell lines: Hct116, UHRR, and SH-SY5Y. The datasets for the Hct116, UHRR, and SH-SY5Y cell lines were sourced from the Singapore Nanopore Expression Project (SG-NEx), the official PacBio website [36], and the Gene Expression Omnibus (GEO) under accession number GSE213984, respectively.

Different genome and transcriptome reference files were used for the analysis of simulated and experimental datasets. For the simulated datasets, we used the GRCh38 (RefSeq 110) human reference genome and transcriptome [35]. For the experimental datasets, we employed the genome file hg38_sequins_SIRV_ERCCs_longSIRVs.fa and the transcriptome file hg38_sequins_SIRV_ERCCs_longSIRVs_cdna.fa, provided by the SG-NEx project which include synthetic SIRV and sequin transcripts in addition to transcripts derived from the GRCh38 reference genome.

To perform alignments of the datasets to both the genome and transcriptome, we utilized the following commands. Commands 2, 3, and 4 correspond to the alignment steps for ONT 1D-cDNA/direct-cDNA sequencing, ONT direct-RNA sequencing, and PacBio datasets to the genome. Commands 5, 6, and 7 represent the steps for aligning ONT, PacBio, and PacBio-HiFi datasets to the transcriptome. In these commands, variables such as `num_threads`, `genome_file`, `transcriptome_file`, `annotation_file`, `input_file`, and `output_file` represent the number of threads, genome file, transcriptome file, annotation file, input FASTQ file, and output BAM file, respectively:

```
1. paftools.js gff2bed annotation_file > bed_file
2. minimap2 --junc-bed bed_file -t num_threads -ax splice -ub --secondary=no genome_file \
   input_file > output_file
3. minimap2 --junc-bed bed_file -t num_threads -ax splice -uf --secondary=no genome_file \
   input_file > output_file
4. minimap2 --junc-bed bed_file -t num_threads -ax splice:hq -k14 -uf --secondary=no \
   genome_file input_file > output_file
5. minimap2 -t num_threads -ax map-ont -N 100 transcriptome_file input_file > output_file
6. minimap2 -t num_threads -ax map-pb -N 100 transcriptome_file input_file > output_file
7. minimap2 -t num_threads -ax map-hifi -N 100 transcriptome_file input_file > output_file
```

In addition, if the downstream quantification tool expects or prefers a `BAM` file rather than a `SAM` file as input, we convert the `SAM` file to a `BAM` file using `samtools`. The conversion command is `samtools view -@ convert_threads -bo output_file.bam output_file.sam`. Further, wherever possible, we prefer to perform the conversion in a streaming fashion rather than to write the `SAM` file and then convert it to `BAM` format. This can be done e.g. by piping the output of `minimap2` directly to the `samtools view` command.

F.2 Quantification Code

We employed **salmon** for quantifying short-read RNA-seq data and obtain the Gibbs sampling results. The procedure involved generating an index file and subsequently utilizing this index to quantify the provided fastq files. The code snippet used for this process is outlined below.

```
1. salmon index -p num_threads -t transcriptome_file -i output_index -k 31
2. salmon quant -p num_threads -i output_index --numGibbsSamples 50 \
   -l A -1 input_file_1 -2 input_file_2 -o output_file
```

We employed two distinct approaches to quantify long reads with **oarfish**: one based on alignment files and the other on raw FASTQ reads. Each approach was implemented in two variations—one incorporating a coverage distribution model and the other without. Specifically, Codes 1 and 2 use alignment files for quantification, while Codes 3 and 4 use raw reads. Codes 1 and 3 incorporate the coverage model, whereas Codes 2 and 4 do not. These codes are outlined below. When processing stranded ONT direct-RNA data, we include the optional argument `-d fw` (shown in brackets), as these reads are expected to align to the forward strand. Additionally, the `--seq-tech` parameter is specified based on the dataset type, with options including: `ont-cdna`, `ont-drna`, `pac-bio`, and `pac-bio-hifi`.

```
1. oarfish --alignments input_bam_file --threads num_threads --output output_directory \
   --filter-group no-filters [-d fw]
2. oarfish --alignments input_bam_file --threads num_threads --output output_directory \
   --model-coverage --filter-group no-filters [-d fw]
3. oarfish --reads fastq_file --reference transcriptome_index --seq-tech sequence_type \
    --threads num_threads --output output_directory --filter-group no-filters [-d fw]
4. oarfish --reads fastq_file --reference transcriptome_index --seq-tech sequence_type \
   --threads num_threads --output output_directory --model-coverage --filter-group no-filters [-d fw]
```

For quantifying the dataset using **bambu**, the following code was employed.

```
1. bambuAnnotations = prepareAnnotations(gtf_annotation)
```

```
2. RcOut1 = bambu(reads = input_bam_file, annotations = bambuAnnotations, genome = genome_file, \
   discovery = FALSE)
```

For quantifying datasets using **NanoCount**, two separate codes were utilized. Code 1 was used for `ONT 1D-cDNA`, `ONT direct-cDNA`, and `PacBio` datasets, while Code 2 was used for `ONT direct-RNA` sequencing datasets. The optional argument which is shown in brackets used only when we want to disable all filters in **NanoCount**.

```
1. NanoCount -i input_bam_file --keep_neg_strand -o output_file [-l 1 -f 0.0001 -d -1]
2. NanoCount -i input_bam_file -o output_file [-l 1 -f 0.0001 -d -1]
```

The subsequent code is employed for quantifying the dataset using **ESPRESSO**.

```
1. samtools sort -@num_threads -O sam input_bam_file > output_sam_sorted
2. echo -e output_sam_sorted"\t sample" > tsv_file
3. perl ESPRESSO_S -A annotation_file -F genome_file -L tsv_file -O output_directory \
   -T num_threads
4. perl ESPRESSO_C -I output_directory -F genome_file -X 0 -T num_threads
5. perl ESPRESSO_Q -A annotation_file -L tsv_file -V output_file -T num_threads
```

To quantify the `ONT` and `PacBio` sequenced datasets with **IsoQuant**, we employed the following code, respectively.

```
1. isoquant.py -d nanopore --fastq fastq_file -t num_threads -g annotation_file \
   -r genome_file --junc_bed_file bed_file --no_model_construction \
   --transcript_quantification all -o output_directory
2. isoquant.py -d pacbio_ccs --fastq fastq_file -t num_threads -g annotation_file \
   -r genome_file --junc_bed_file bed_file --no_model_construction \
   --transcript_quantification all -o output_directory
```

For quantification with **lr-kallisto**, Code 1 was used only for ONT direct-RNA sequencing datasets. Conversion of `u/U` bases to `t/T` is necessary for quantifying ONT direct-RNA data. The files generated during the run, including `output.bus`, `transcripts.txt`, `matrix.ec`, `count.mtx`, and `flens.txt`, are utilized in subsequent commands. The file `LRGASP_t2g` is generated from the `annotation_file` and lists transcripts with their corresponding genes. The final code uses different `-P` parameters based on whether ONT or PacBio technology was used.

```
1. perl -pe 'tr/uU/tT/ unless(/@+/)' < drna_fastq_file | gzip > fastq_file
2. kallisto index -k 63 -i index_file transcriptome_file -t num_threads
3. kallisto bus -t num_threads -x bulk --long --threshold 0.8 -i index_file fastq_file \
   -o output_directory
4. bustools sort -t num_threads output.bus -o sorted_bus_file
5. bustools count sorted_bus_file -t transcripts.txt -e matrix.ec -o bustool_quant --cm -m \
   -g LRGASP_t2g
6. kallisto quant-tcc -t num_threads --long -P ONT/PacBio count.mtx \
   -f flens.txt -i index_file -e matrix.ec -o output_directory
```

To quantify the dataset using **TranSigner**, which consists of three distinct modules—align, prefilter, and em—we used the following commands. Code 1 is employed for aligning the `ONT` dataset, with the optional argument (in brackets) applied when aligning `PacBio` datasets. Codes 2 and 3 are used for the prefilter step: Code 2 is specifically for the noisy `ONT direct-RNA` sequencing dataset, while Code 3 is used for the `ONT 1D-cDNA/direct-cDNA` and `PacBio` sequencing datasets. Finally, Code 4 is used for the em step, which is common to all sequencing datasets. During the prefilter step, the `scores.tsv` and `ti.pkl` files are generated.

```
1. transigner align -q fastq_file -t transcriptome_file -d output_directory -o bam_file \
   -p num_threads -sN 100 -v [-mm2=\"-ax map-hifi\"]
2. transigner prefilter -a bam_file -t transcriptome_file -o output_directory --filter -tp -1
3. transigner prefilter -a bam_file -t transcriptome_file -o output_directory --filter \
   -tp -500 -fp -600
4. transigner em -s scores.tsv -i ti.pkl -o output_directory --drop --use-score
```

G Supplementary Tables

Table S1. Evaluation metrics on the 1D cDNA (ONT) and RSII (PacBio) simulation reads obtained from H9 and UHRR cell lines for the evaluated methods.

Method	Spearman ρ		Pearson ($\log(1+x)$)		CCC		Kendall-τ		RMSE		NRMSE		MARD	
	cDNA	RSII	cDNA	RSII	cDNA	RSII	cDNA	RSII	cDNA	RSII	cDNA	RSII	cDNA	RSII
`oarfish` (cov)	**0.91**	**0.94**	**0.97**	**0.98**	**0.97**	**0.98**	**0.89**	**0.92**	**73.92**	**79.75**	**1.11**	**1.01**	**0.06**	**0.06**
`oarfish` (nocov)	0.87	0.93	**0.97**	**0.98**	**0.97**	**0.98**	0.85	0.91	237.37	142.20	3.56	1.81	0.08	**0.06**
`NanoCount`	0.57	0.66	0.74	0.79	0.67	0.78	0.51	0.58	1118.02	789.02	16.76	10.02	0.35	0.39
`NanoCount` (nofilt)	0.65	0.73	0.88	0.92	0.88	0.92	0.57	0.65	530.92	285.13	7.96	3.62	0.41	0.33
`bambu`	0.82	0.87	0.92	0.94	0.92	0.94	0.79	0.84	609.98	186.31	9.14	2.37	0.11	0.11
`lr-kallisto`	0.73	0.65	0.91	0.77	0.91	0.71	0.69	0.60	491.51	1111.39	7.37	14.12	0.17	0.25
`TranSigner`	0.64	0.71	0.86	0.91	0.85	0.90	0.56	0.63	363.78	103.75	5.45	1.32	0.46	0.39
`IsoQuant`	0.88	0.89	0.93	0.92	0.93	0.92	0.85	0.85	565.76	434.74	8.48	5.52	0.08	0.11
`ESPRESSO`	0.80	0.83	0.85	0.90	0.77	0.85	0.77	0.80	1121.71	949.41	16.82	12.06	0.14	0.16

Table S2. Evaluation metrics on the 1D cDNA (ONT) and direct-RNA (ONT) simulation reads obtained from NA12878 sample for the evaluated methods.

Method	Spearman ρ		Pearson ($\log(1+x)$)		CCC		Kendall-τ		RMSE		NRMSE		MARD	
	cDNA	dRNA	cDNA	dRNA	cDNA	dRNA	cDNA	dRNA	cDNA	dRNA	cDNA	dRNA	cDNA	dRNA
`oarfish` (cov)	**0.89**	**0.91**	**0.96**	**0.98**	**0.96**	**0.98**	**0.87**	**0.90**	**72.09**	**46.09**	**1.32**	**0.95**	**0.06**	**0.04**
`oarfish` (nocov)	0.84	0.87	**0.96**	**0.98**	**0.96**	**0.98**	0.82	0.86	227.45	147.21	4.18	3.03	0.09	0.06
`NanoCount`	0.53	0.52	0.70	0.73	0.59	0.67	0.48	0.47	1173.03	814.03	21.55	16.78	0.32	0.32
`NanoCount` (nofilt)	0.63	0.59	0.89	0.84	0.88	0.83	0.56	0.52	366.42	318.90	6.73	6.57	0.37	0.36
`bambu`	0.79	0.80	0.90	0.91	0.90	0.91	0.76	0.77	428.25	374.29	7.87	7.71	0.12	0.10
`lr-kallisto`	0.65	0.64	0.87	0.87	0.86	0.87	0.61	0.61	666.61	587.64	12.25	12.11	0.20	0.18
`TranSigner`	0.61	0.57	0.84	0.81	0.82	0.79	0.54	0.50	274.59	240.27	5.04	4.95	0.42	0.44
`IsoQuant`	0.84	0.89	0.90	0.93	0.90	0.93	0.82	0.87	443.04	353.98	8.14	7.29	0.08	0.06
`ESPRESSO`	0.72	0.82	0.78	0.87	0.66	0.80	0.69	0.80	1213.71	875.25	22.30	18.04	0.14	0.10

Table S3. Evaluation metrics on the direct-RNA (ONT) and direct-cDNA (ONT) experimental reads obtained from Hct116 cell line for the evaluated methods, based only on the sequin spike-in transcripts.

Method	Spearman ρ		Pearson ($\log(1+x)$)		CCC		Kendall-τ		RMSE		NRMSE		MARD	
	cDNA	dRNA	cDNA	dRNA	cDNA	dRNA	cDNA	dRNA	cDNA	dRNA	cDNA	dRNA	cDNA	dRNA
`oarfish` (cov)	**0.92**	**0.62**	**0.96**	**0.70**	**0.94**	**0.56**	**0.81**	**0.52**	10328.92	**10014.90**	**1.69**	**1.64**	**0.62**	**0.87**
`oarfish` (nocov)	**0.92**	**0.62**	**0.96**	**0.70**	**0.94**	**0.56**	**0.81**	**0.52**	**10316.47**	10026.93	**1.69**	**1.64**	**0.62**	**0.87**
`NanoCount`	0.91	**0.62**	0.94	0.69	0.92	**0.56**	0.80	**0.52**	16384.78	14091.06	2.69	2.31	0.65	**0.87**
`bambu`	**0.92**	0.60	0.95	0.66	0.93	0.53	**0.81**	0.49	10417.71	22311.14	1.71	3.66	0.63	0.88
`lr-kallisto`	0.88	0.46	0.93	0.51	0.90	0.37	0.76	0.38	11627.02	23986.10	1.91	3.93	0.66	0.93
`TranSigner`	0.90	0.61	0.95	0.69	0.93	**0.56**	0.79	0.50	10337.42	10162.80	1.70	1.67	**0.62**	**0.87**
`IsoQuant`	0.89	0.60	0.93	0.67	0.92	0.53	0.78	0.50	11946.23	19082.30	1.96	3.13	**0.62**	0.88
`ESPRESSO`	0.89	0.58	0.93	0.64	0.91	0.50	0.77	0.48	17610.95	20950.32	2.89	3.44	0.65	0.89

H Supplementary Figures

H.1 Illustrative Figures for Methodology and Conceptual Clarification

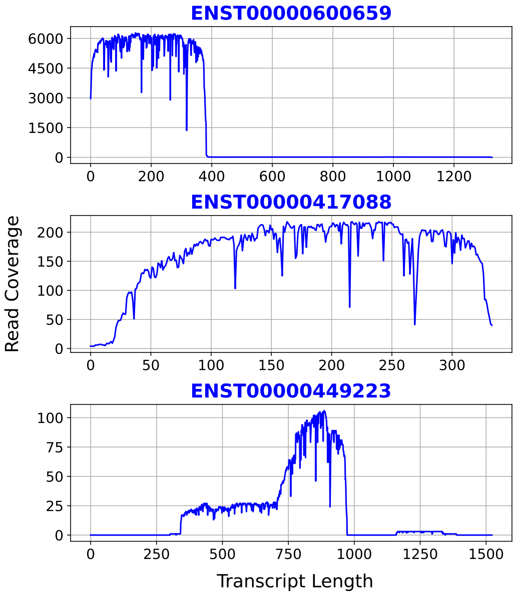

Supplementary Figure S1. The coverage distribution model for three transcripts ENST00000600659, ENST00000417088, and ENST00000449223, as estimated from the BAM file of the Hct116 cell line dataset sequenced with direct cDNA.

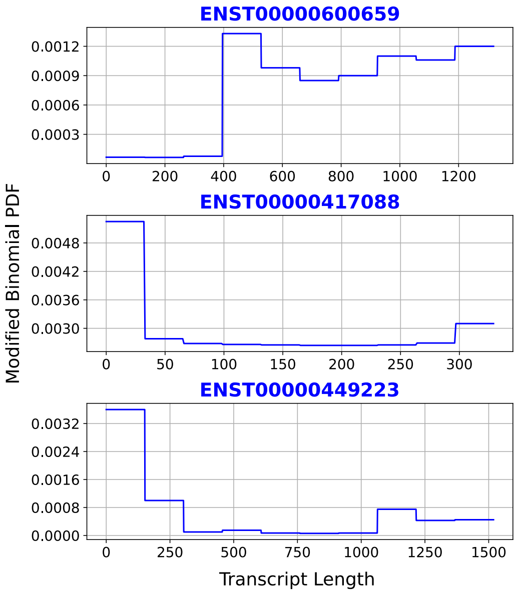

Supplementary Figure S2. Coverage assignment probability distribution for three transcripts ENST00000600659, ENST00000417088, and ENST00000449223 from the Hct116 dataset BAM file.

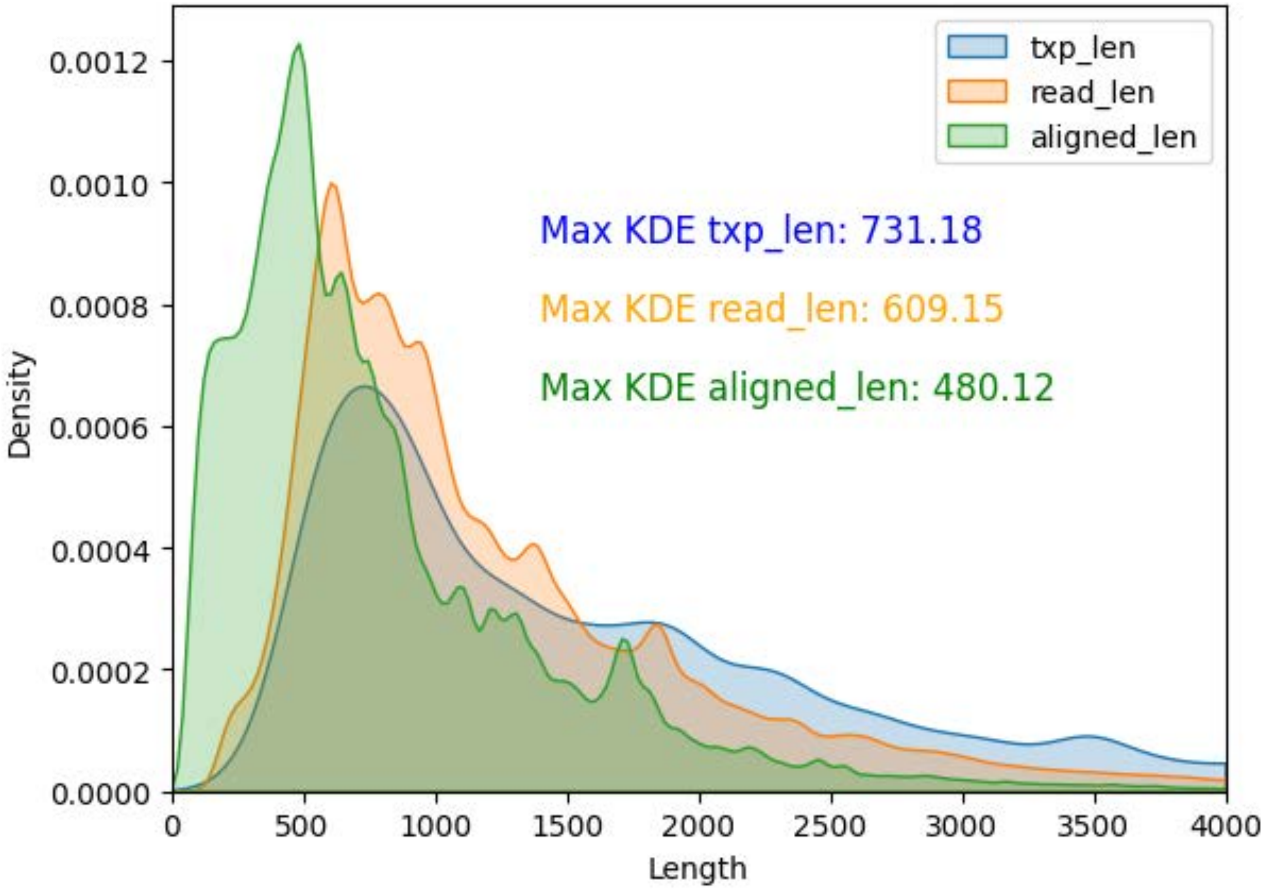

Supplementary Figure S3. Transcript length, read length, and aligned part of the read length density distribution. This figure illustrates the density distribution of transcript length (`txp_len`), read length (`read_len`), and aligned length of reads to transcripts (`aligned_len`). The analysis is conducted using a long-read RNA-seq dataset from the Hct116 cell line, sequenced with the direct-cDNA protocol. The length distribution is depicted up to a maximum length of 4000.

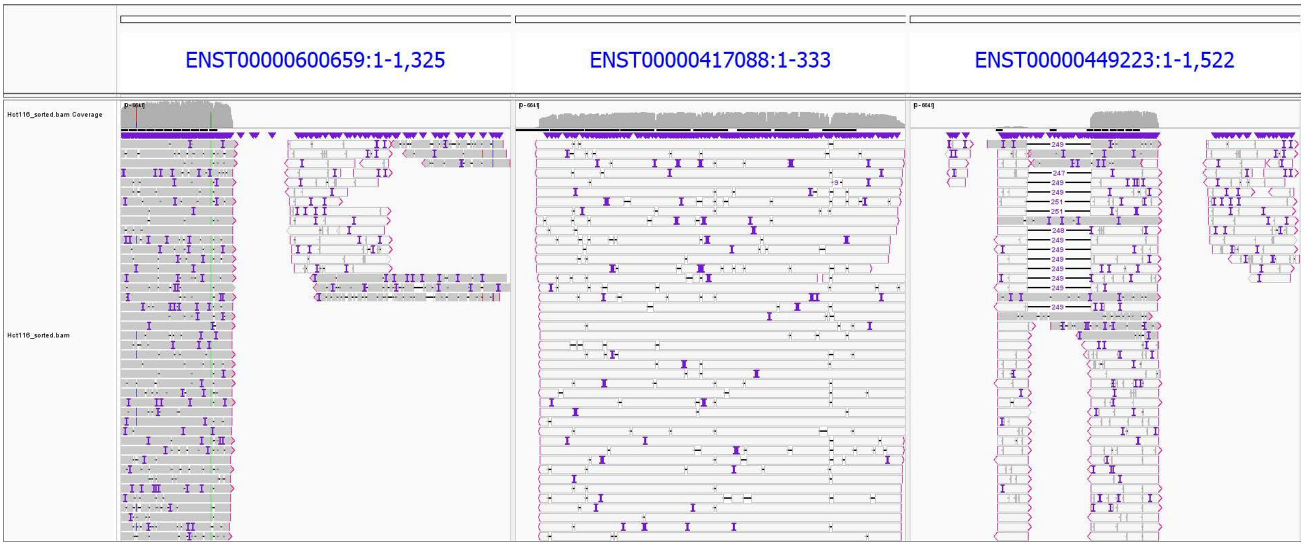

Supplementary Figure S4. The output of IGV tools for three transcripts `ENST00000600659`, `ENST00000417088`, and `ENST00000449223` from the BAM file of the Hct116 cell line dataset sequenced with direct-cDNA.

H.2 Simulated Datasets: Correlation & Error Metrics

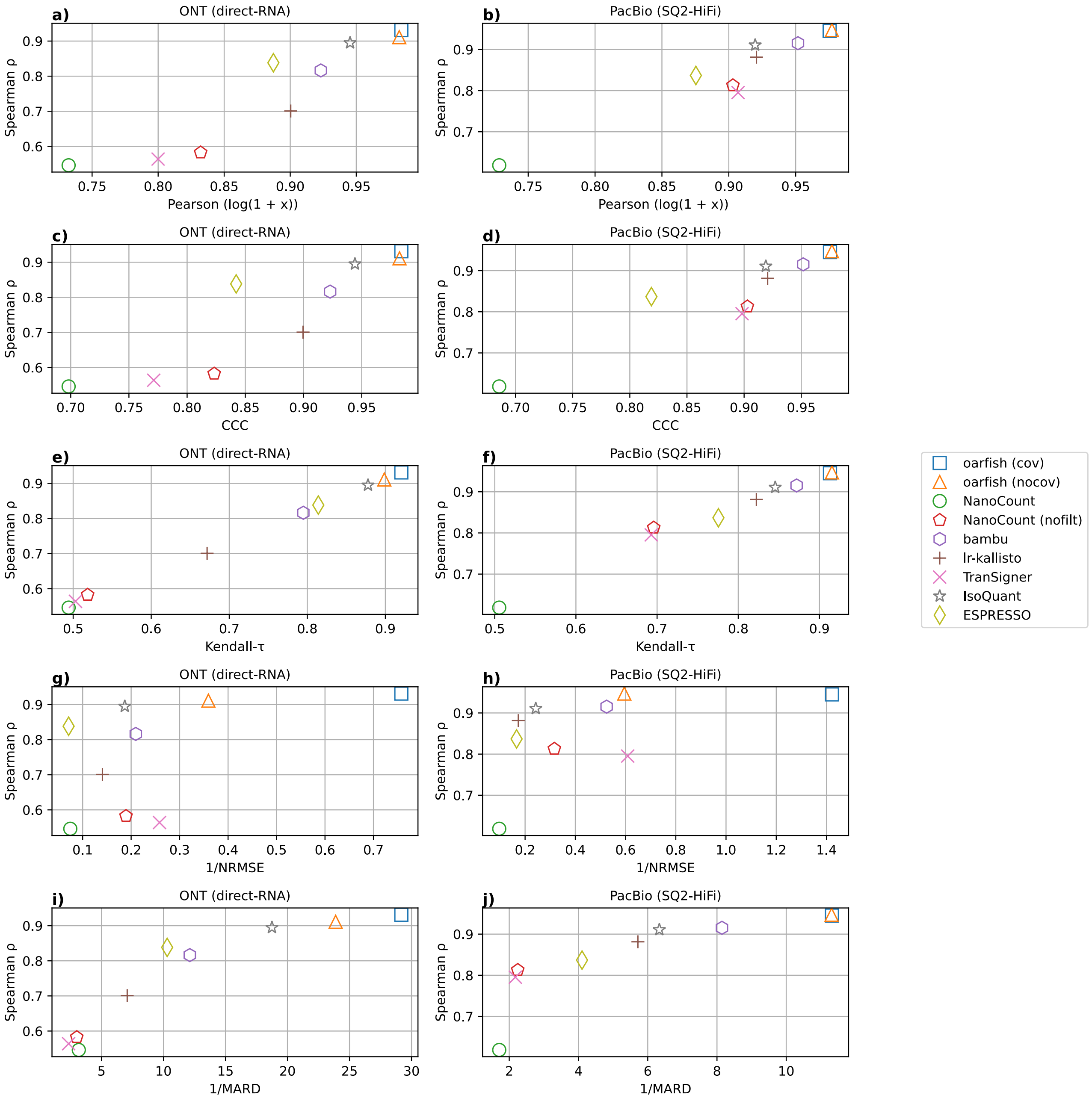

Supplementary Figure S5. compare metrics for both `direct-RNA` and `SQ2-HiFi` datasets sequenced from **H9 & UHRR cell line** samples.

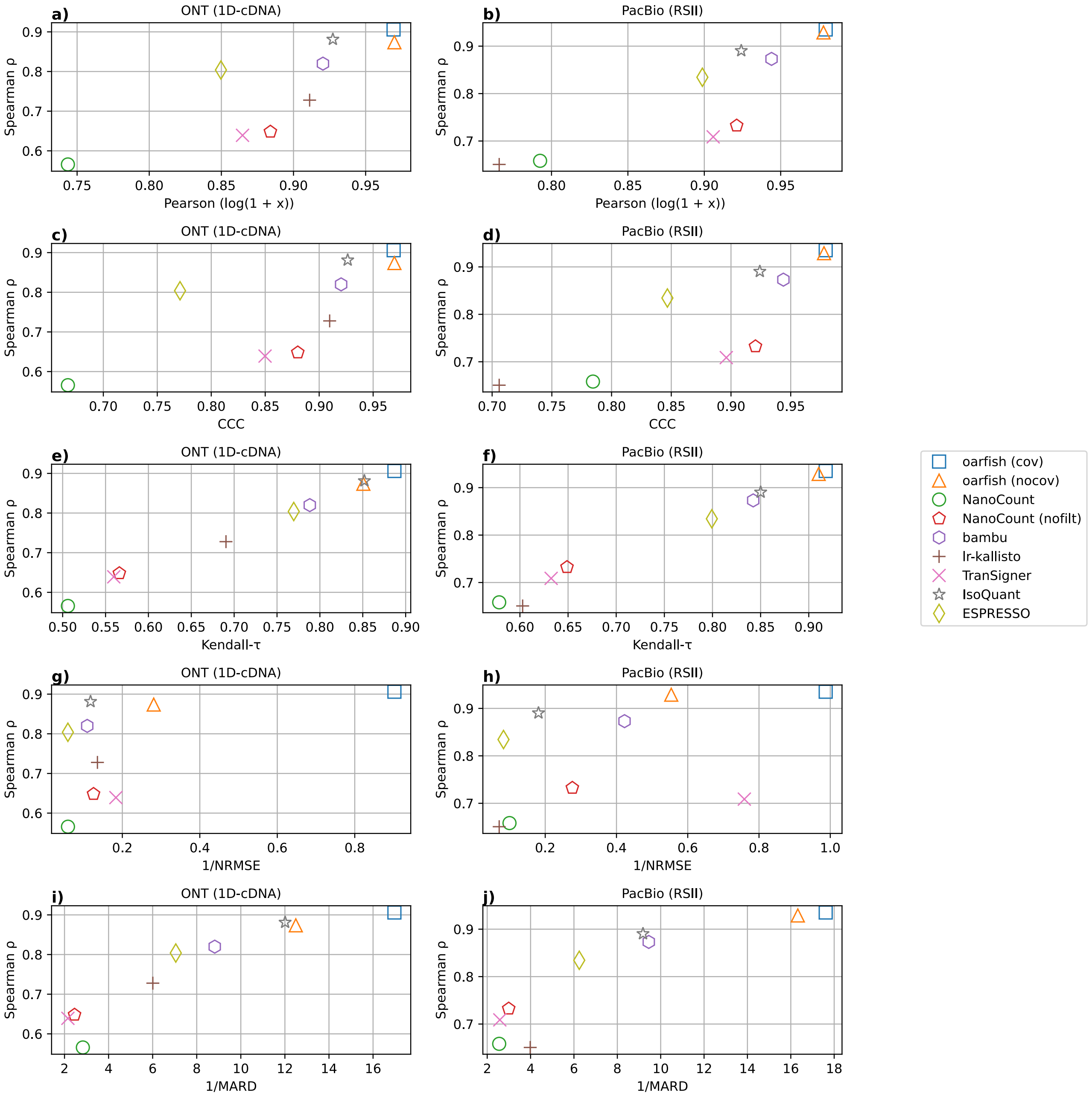

Supplementary Figure S6. compare metrics for both `1D-cDNA` and `RSII` datasets sequenced from **H9 & UHRR cell line** samples.

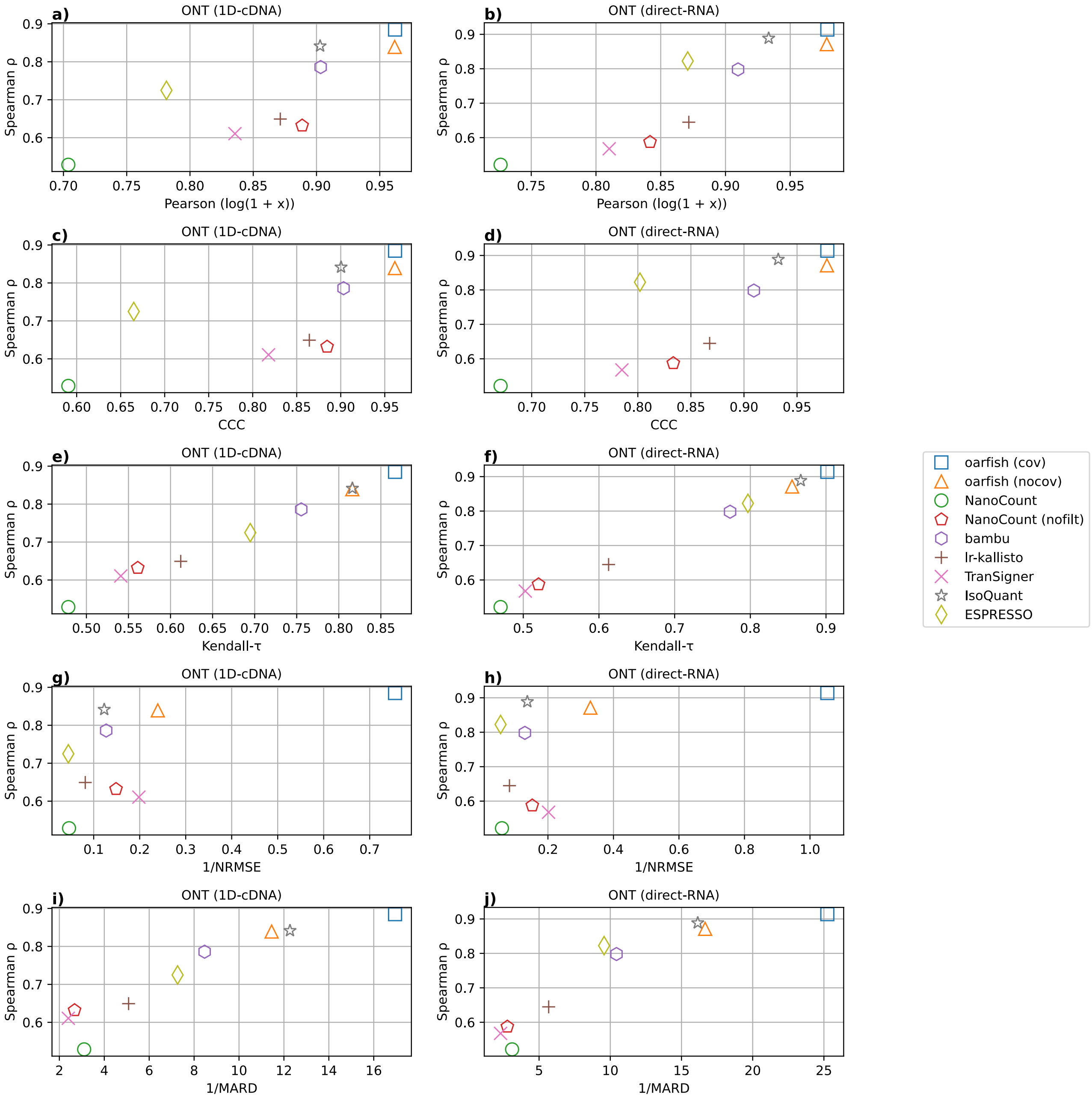

Supplementary Figure S7. compare metrics for both `1D-cDNA` and `direct-RNA` datasets sequenced from **NA12878** sample.

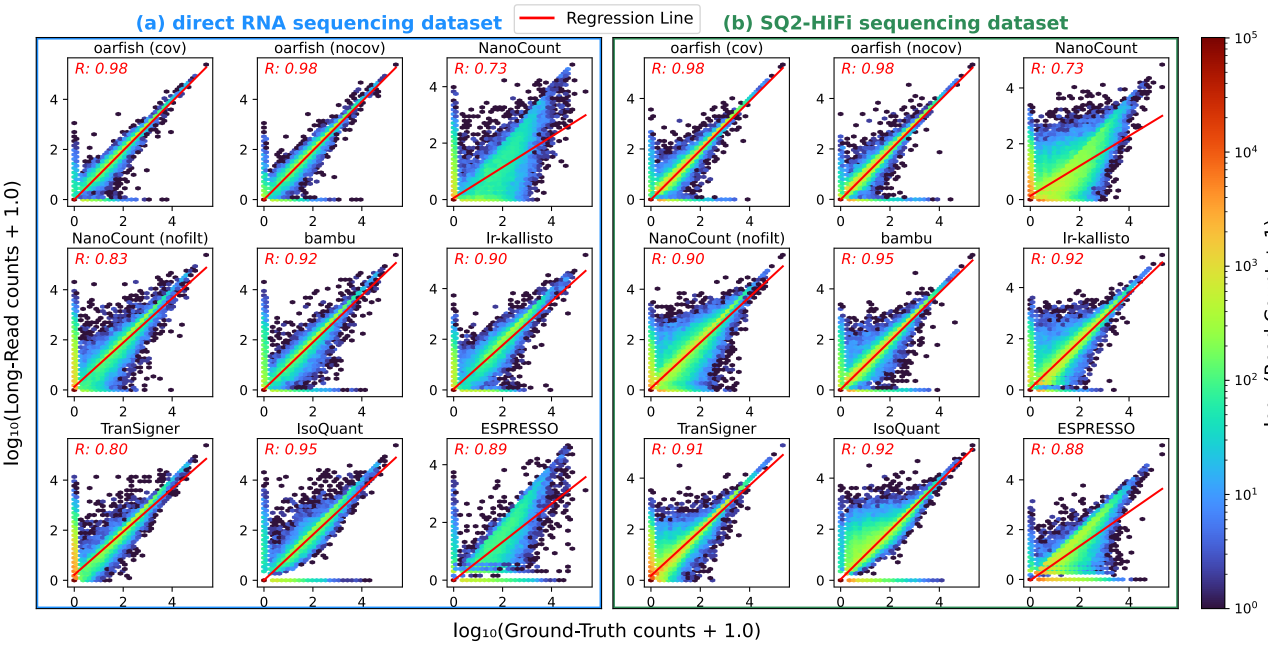

Supplementary Figure S8. density plot for simulated long read counts obtained from H9 & UHRR cell lines and their corresponding ground truth on all transcripts. In all of these methods, the p-value for the Pearson correlation is almost zero (P-value $\simeq$0.0). (a) The ONT long read RNA-seq dataset sequenced with `direct-RNA` protocol from H9 cell line sample. (b) The PacBio long read RNA-seq dataset sequenced with `SQ2-HiFi` protocol from UHRR cell line sample.

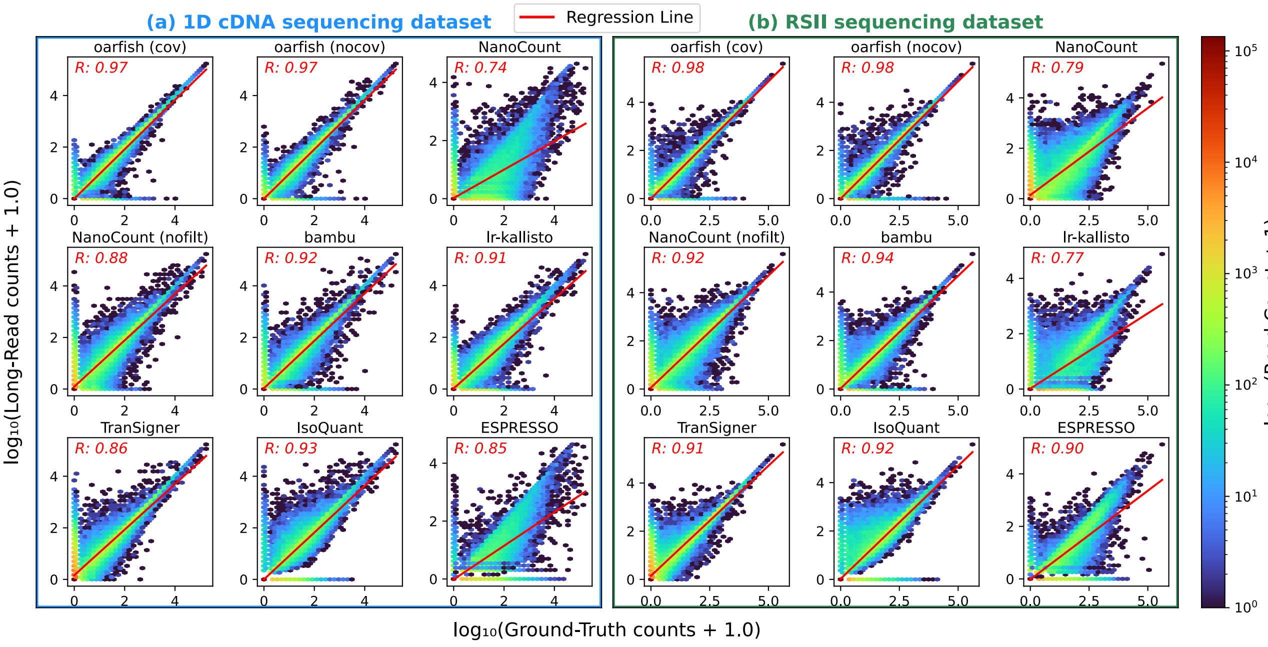

Supplementary Figure S9. density plot for simulated long read counts obtained from **H9 & UHRR cell lines** and their corresponding ground truth on all transcripts. In all of these methods, the p-value for the Pearson correlation is almost zero (P-value $\simeq$0.0). (a) The ONT long read RNA-seq dataset sequenced with `1D-cDNA` protocol from **H9 cell line**. (b) The PacBio long read RNA-seq dataset sequenced with `RSII` protocol from **UHRR cell line**.

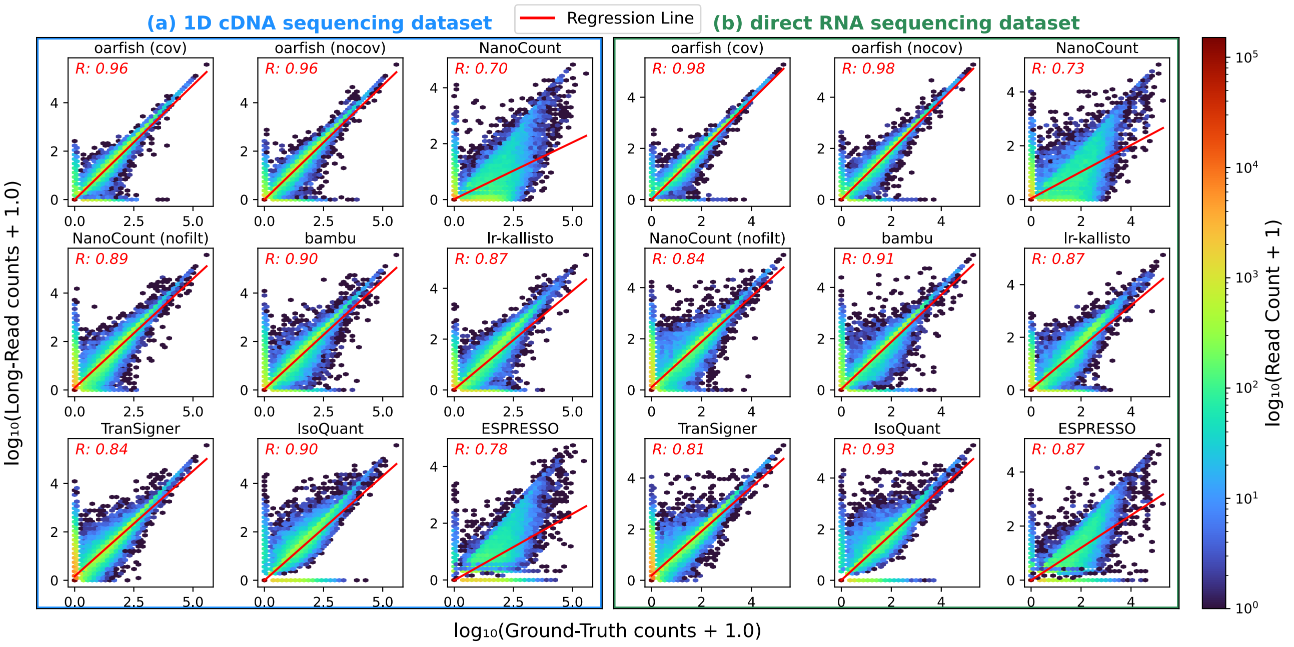

Supplementary Figure S10. density plot for simulated long read counts obtained from the **NA12878 sample** and their corresponding ground truth on all the transcripts. In all of these methods, the p-value for the Pearson correlation is almost zero (P-value $\simeq$0.0). (a) The ONT long read RNA-seq dataset sequenced with `1D-cDNA` protocol. (b) The ONT long read RNA-seq dataset sequenced with `direct-RNA` protocol.

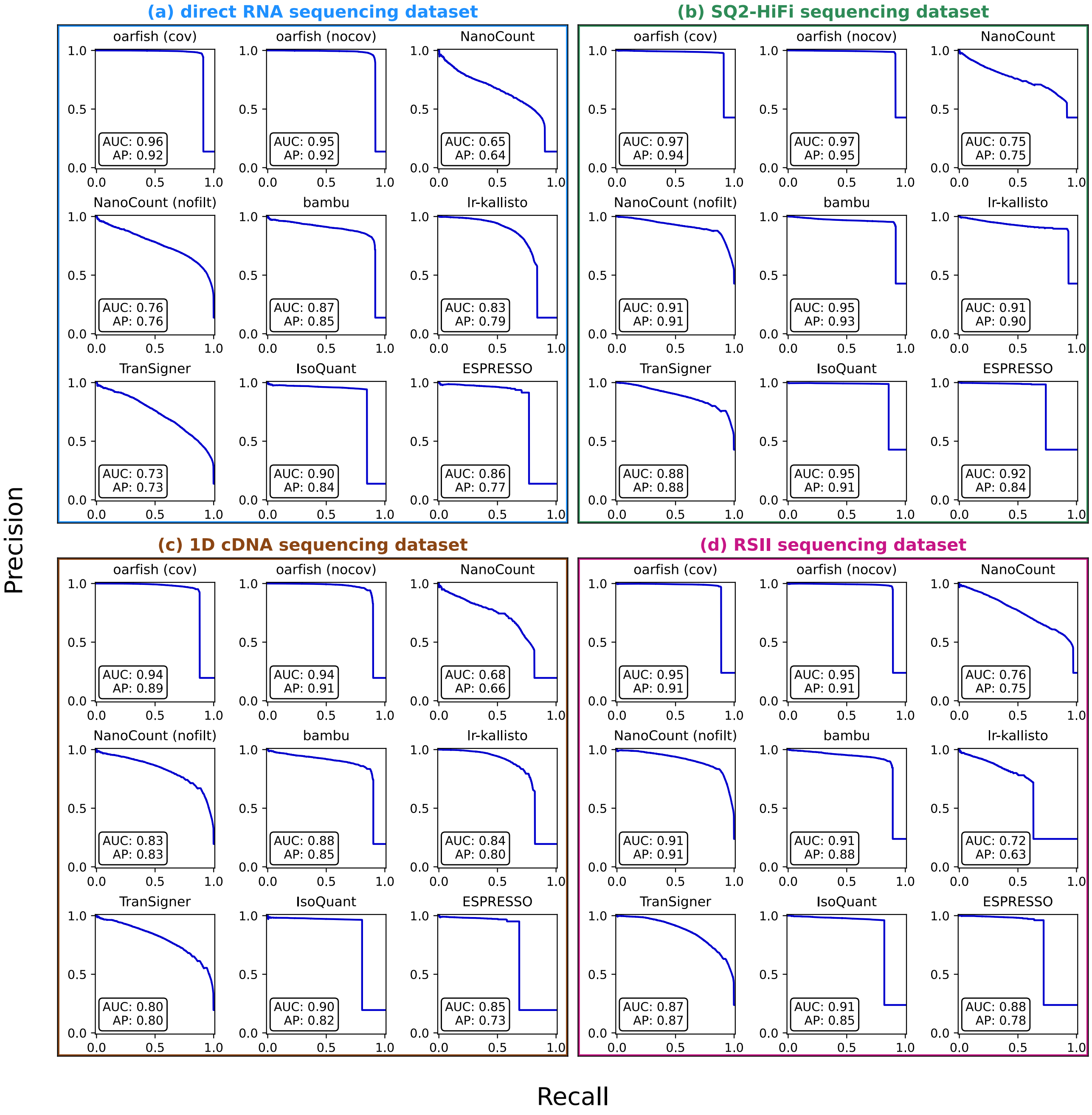

Supplementary Figure S11. precision-recall curve for simulated long read counts obtained from the **H9 & UHRR cell lines** and their corresponding ground truth on all the transcripts. (a) The ONT long read RNA-seq dataset sequenced with `direct-RNA` protocol from **H9 cell line**. (b) The PacBio long read RNA-seq dataset sequenced with `SQ2-HiFi` protocol from **UHRR cell line**. (c) The ONT long read RNA-seq dataset sequenced with `1D-cDNA` protocol from **H9 cell line**. (d) The PacBio long read RNA-seq dataset sequenced with `RSII` protocol from **UHRR cell line**.

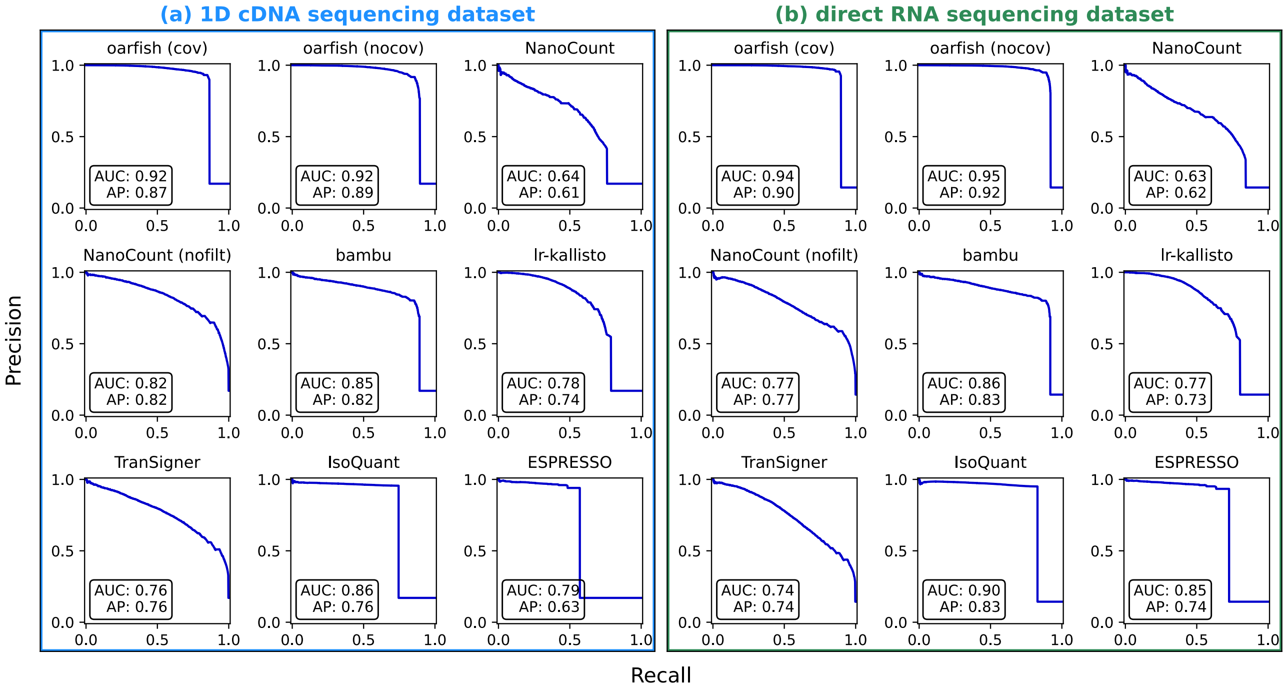

Supplementary Figure S12. precision-recall curve for simulated long read counts obtained from the **NA12878 sample** and their corresponding ground truth on all the transcripts. (a) The ONT long read RNA-seq dataset sequenced with `1D-cDNA` protocol. (b) The ONT long read RNA-seq dataset sequenced with `direct-RNA` protocol.

H.3 Simulated Dataset: Runtime & Memory Usage

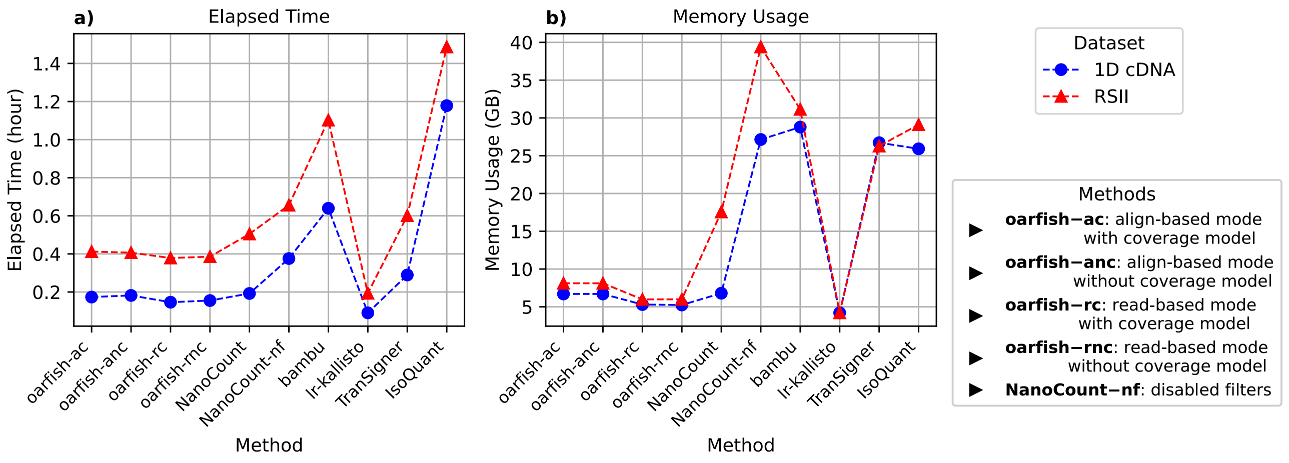

Supplementary Figure S13. performance metrics (elapsed time and peak memory usage) for the proposed and alternative methods using the `1D-cDNA` and `RSII` simulated datasets from **H9 & UHRR cell lines**, respectively: (a) Elapsed time for all methods presented in hours. (b) Memory usage for all methods displayed in Gigabytes. In these figures, `1D-cDNA` and `RSII` represent ONT long reads sequenced with `1D-cDNA` protocol and PacBio long reads sequenced with `RSII` protocol, respectively.

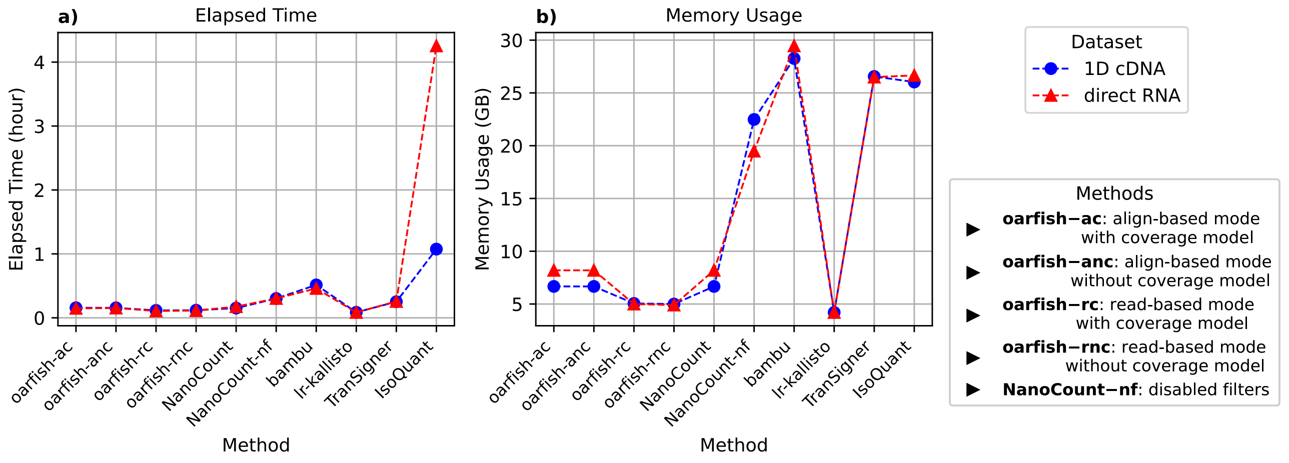

Supplementary Figure S14. Performance metrics (elapsed time and peak memory usage) for the proposed and alternative methods using the `1D-cDNA` and `direct-RNA` simulated datasets from the **NA12878 sample**: (a) Elapsed time for all methods presented in hours. (b) Memory usage for all methods displayed in Gigabytes. In these figures, `1D-cDNA` and `direct-RNA` represent ONT long reads sequenced with `1D-cDNA` protocol and `direct-RNA` protocol, respectively.

H.4 Experimental Datasets (Hct116 & UHRR): Correlation & Error Metrics

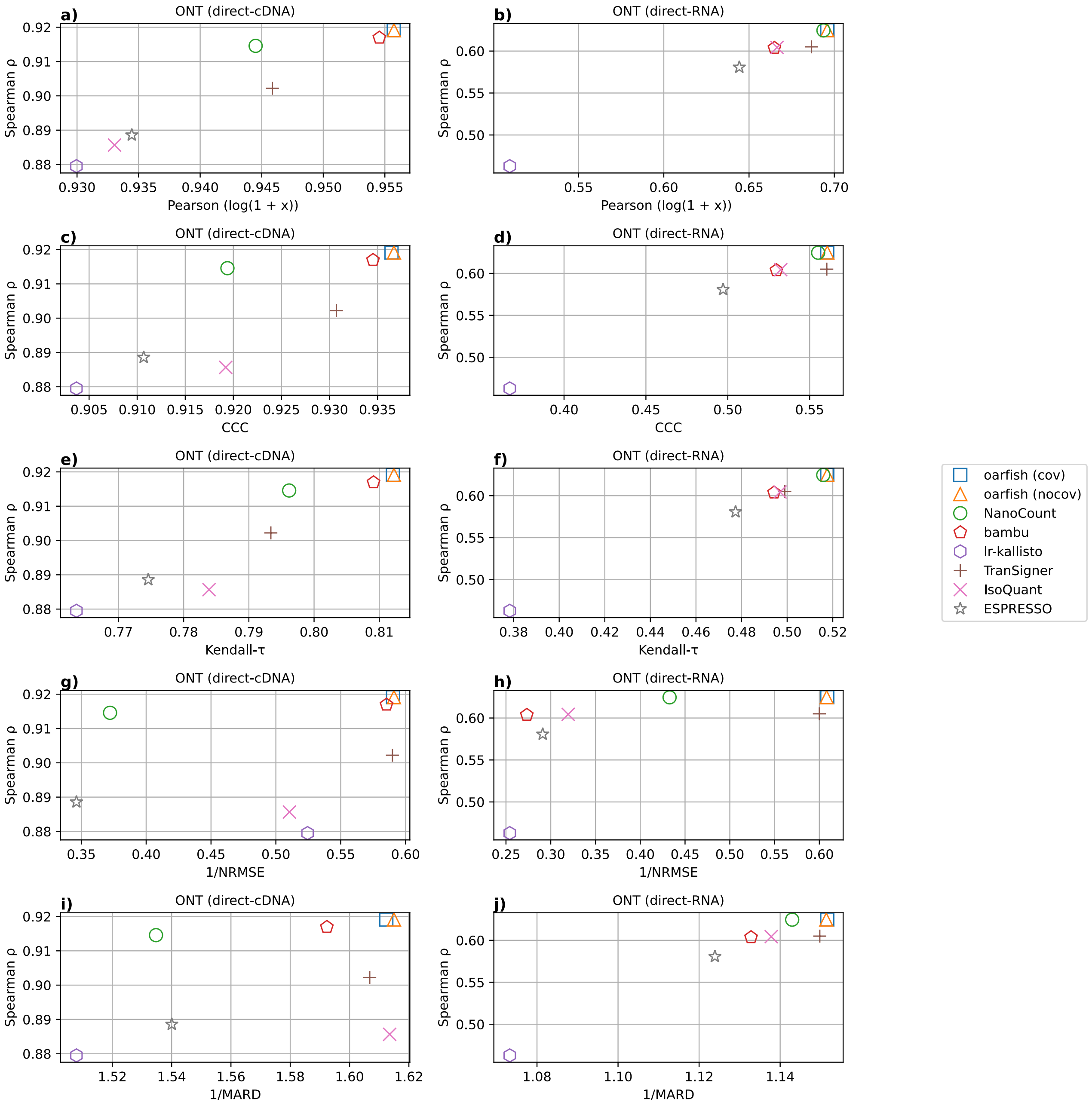

Supplementary Figure S15. compare metrics for both `direct-cDNA` and `direct-RNA` datasets sequenced from the **Hct116 cell line** sample.

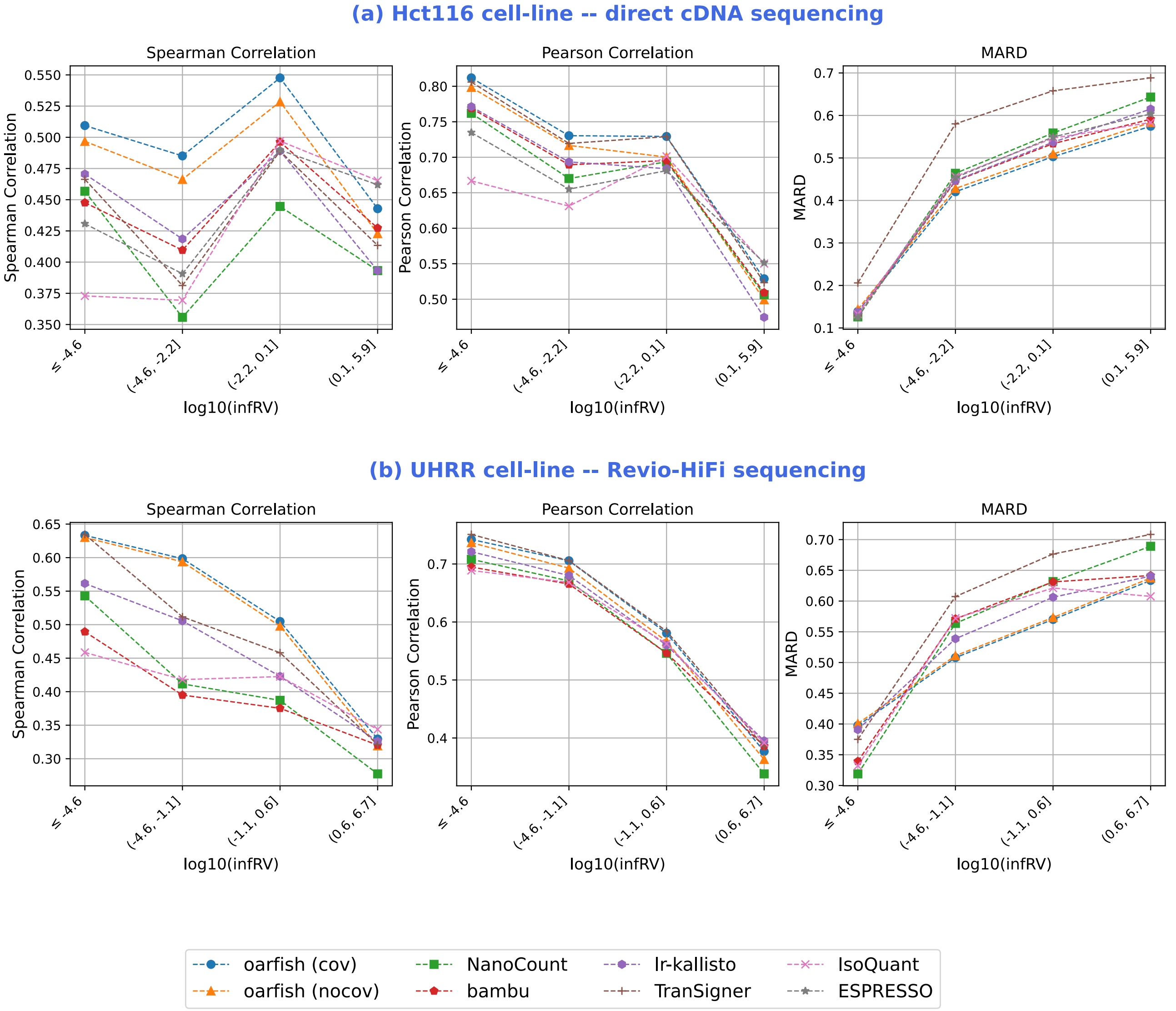

Supplementary Figure S16. Spreaman correlation, Pearson correlation, and MARD between long read and short read quantification results on different subset of InfRV values on all the transcripts. (a) The correlation and error metrics on ONT long read RNA-seq dataset sequenced with `direct-cDNA` protocol from the **Hct116 cell line**. (b) The correlation and error metrics on PacBio long read RNA-seq dataset sequenced with `Revio-HiFi` protocol from the **UHRR sample**.

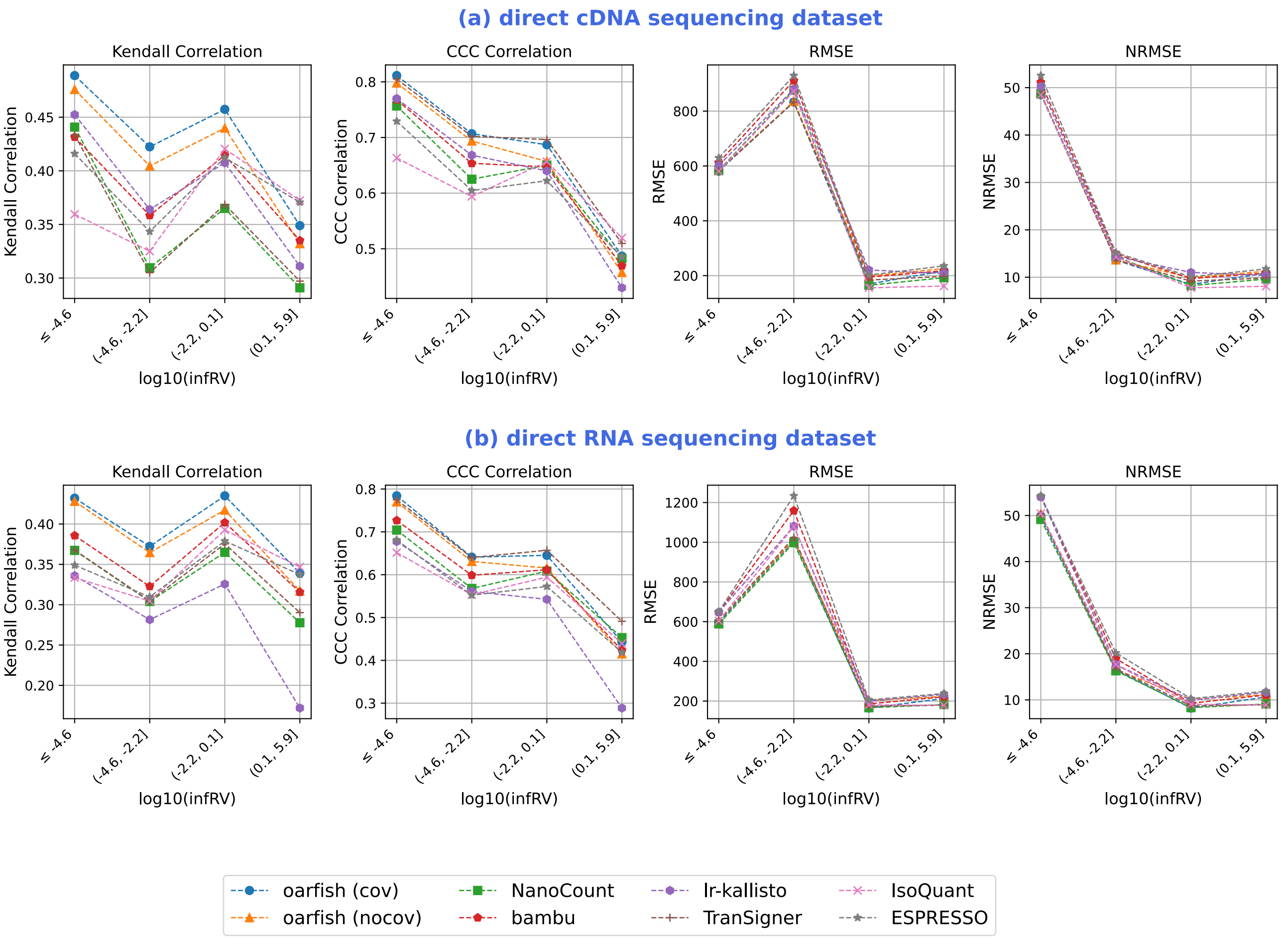

Supplementary Figure S17. Kendall correlation, CCC correlation, RMSE and NRMSE between long read and short read quantification results on different subset of InfRV values on all the transcripts for **Hct116 cell line** dataset. (a) The correlation and error metrics ONT long read RNA-seq dataset sequenced with `direct-cDNA` protocol. (b) The correlation and error metrics ONT long read RNA-seq dataset sequenced with `direct-RNA` protocol.

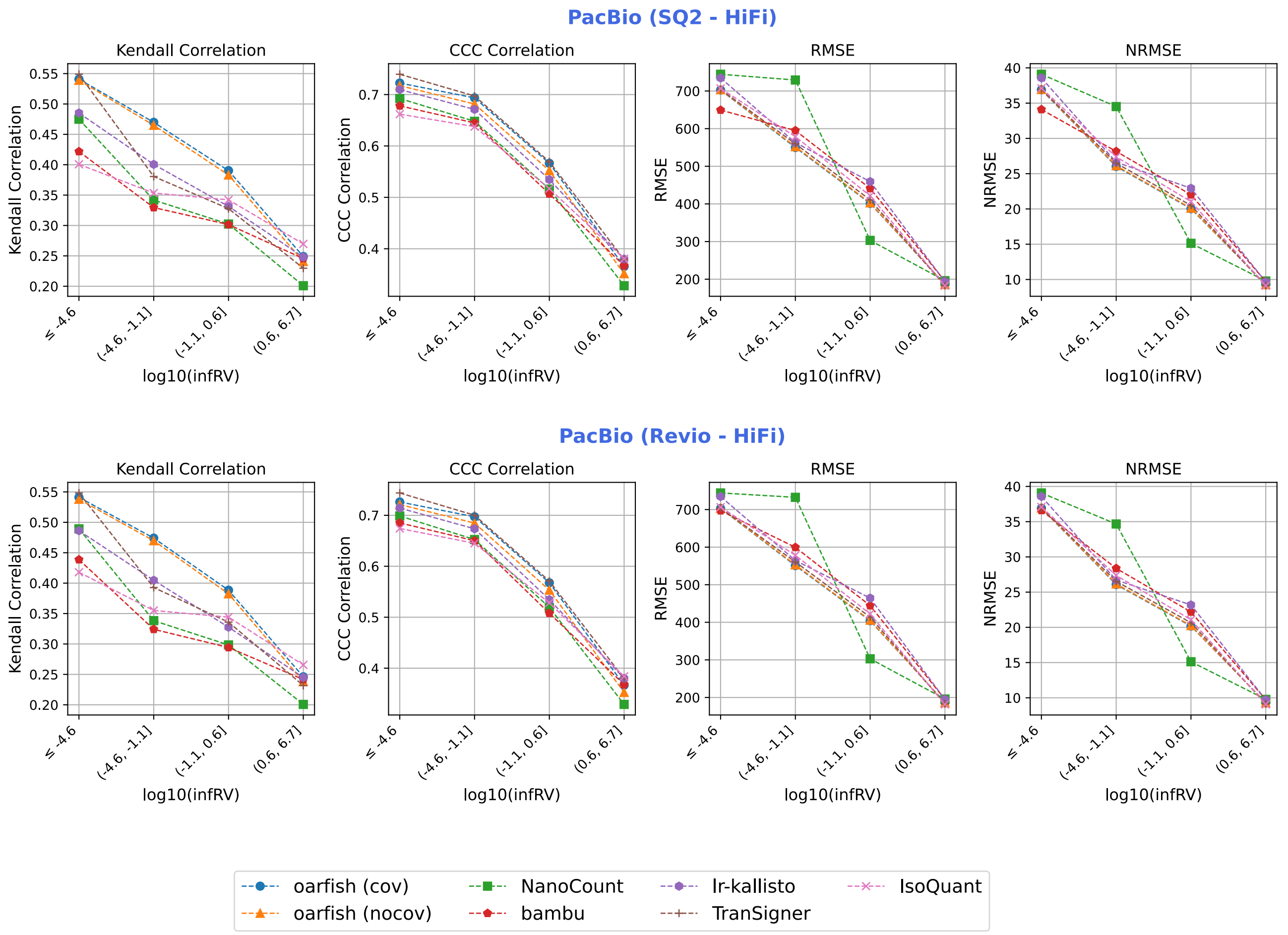

Supplementary Figure S18. Kendall correlation, CCC correlation, RMSE and NRMSE between long read and short read quantification results on different subset of InfRV values on all the transcripts for the **UHRR** dataset. (a) The correlation and error metrics PacBio long read RNA-seq dataset sequenced with `SQ2-HiFi` protocol. (b) The correlation and error metrics PacBio long read RNA-seq dataset sequenced with `Revio-HiFi` protocol.

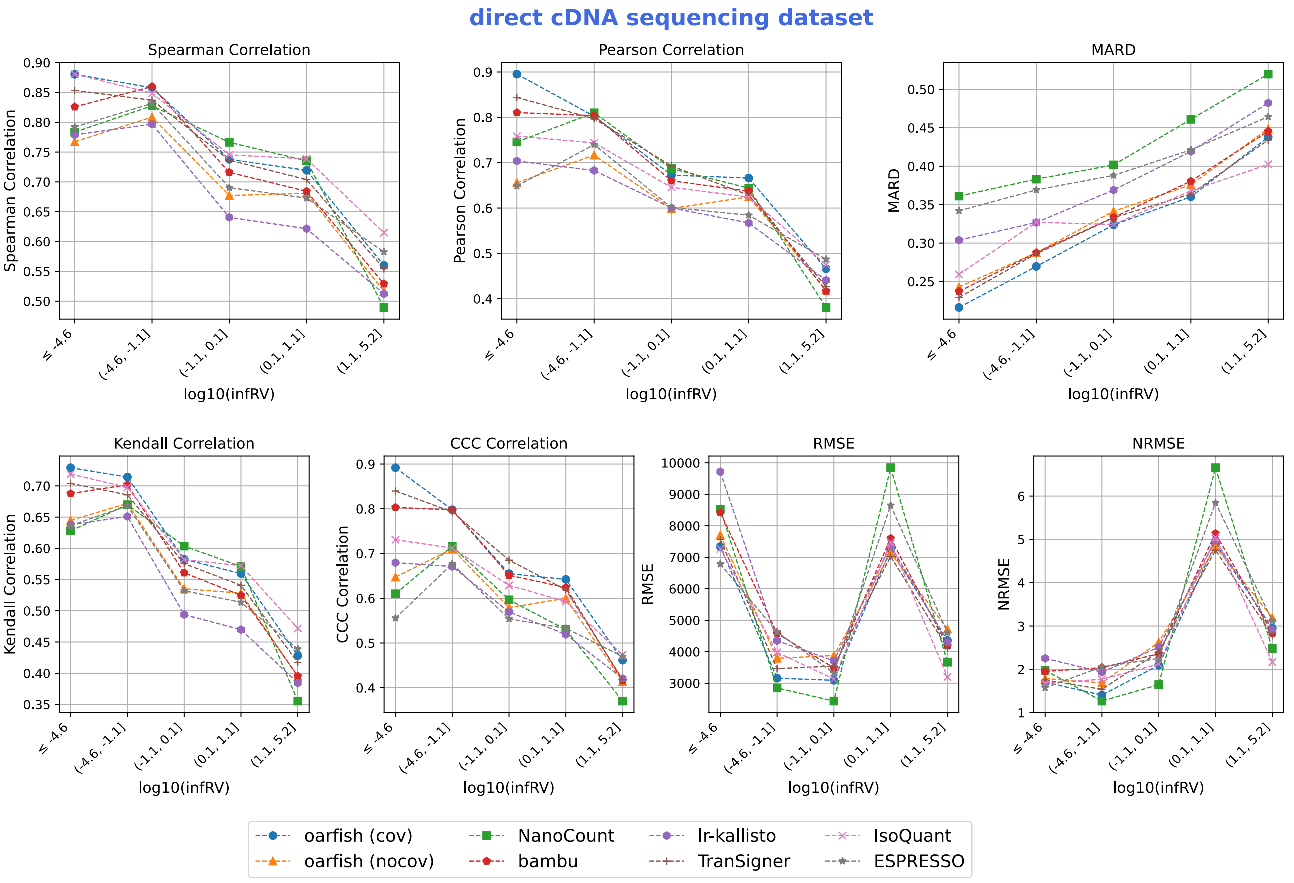

Supplementary Figure S19. Spreaman correlation, Pearson correlation, Kendall correlation, CCC, RMSE, NRMSE and MARD between long read and short read quantification results on different subset of InfRV values on only the major transcripts for **Hct116 cell line** dataset sequenced with `direct-cDNA` protocol.

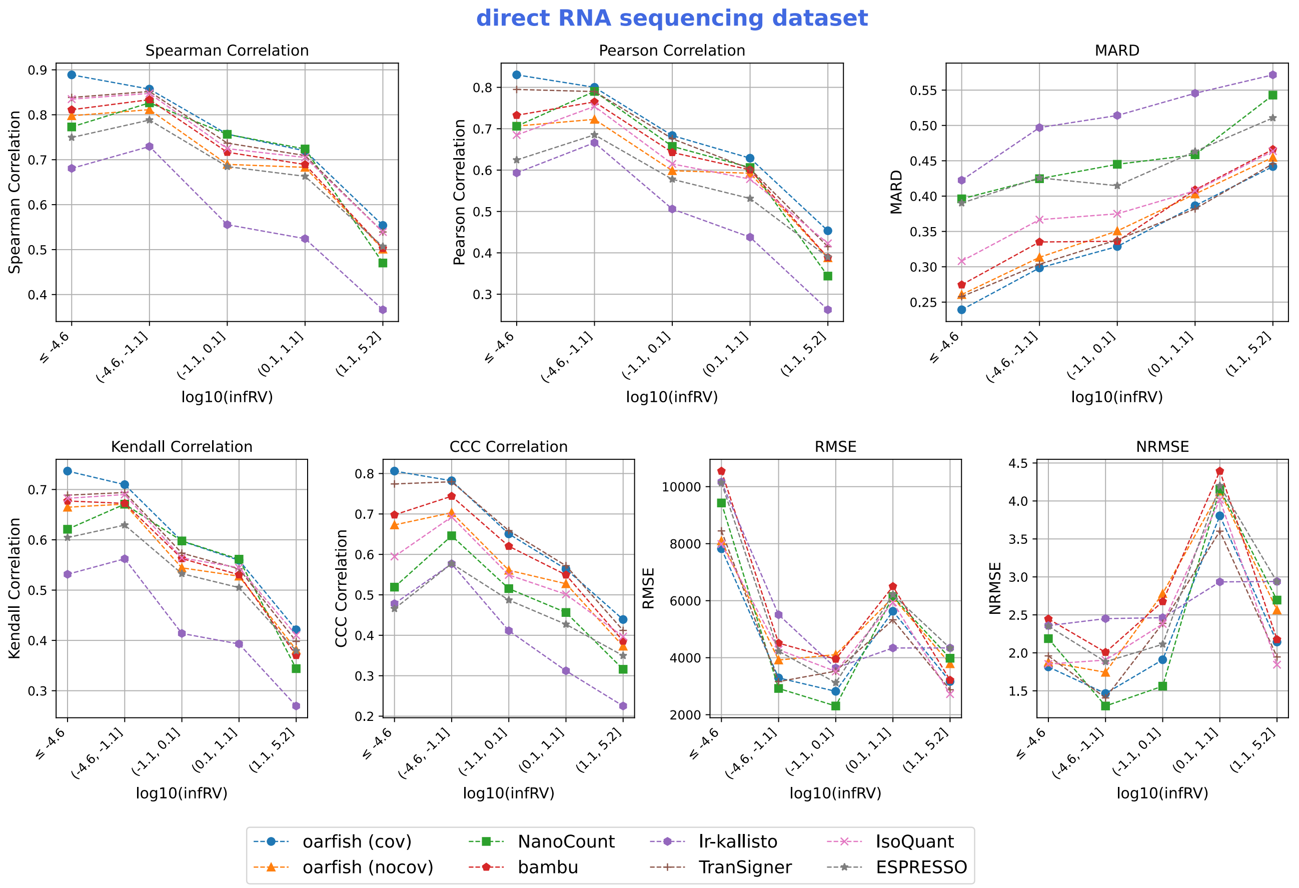

Supplementary Figure S20. Spreaman correlation, Pearson correlation, Kendall correlation, CCC, RMSE, NRMSE and MARD between long read and short read quantification results on different subset of InfRV values on only the major transcripts for the **Hct116 cell line** dataset sequenced with `direct-RNA` protocol.

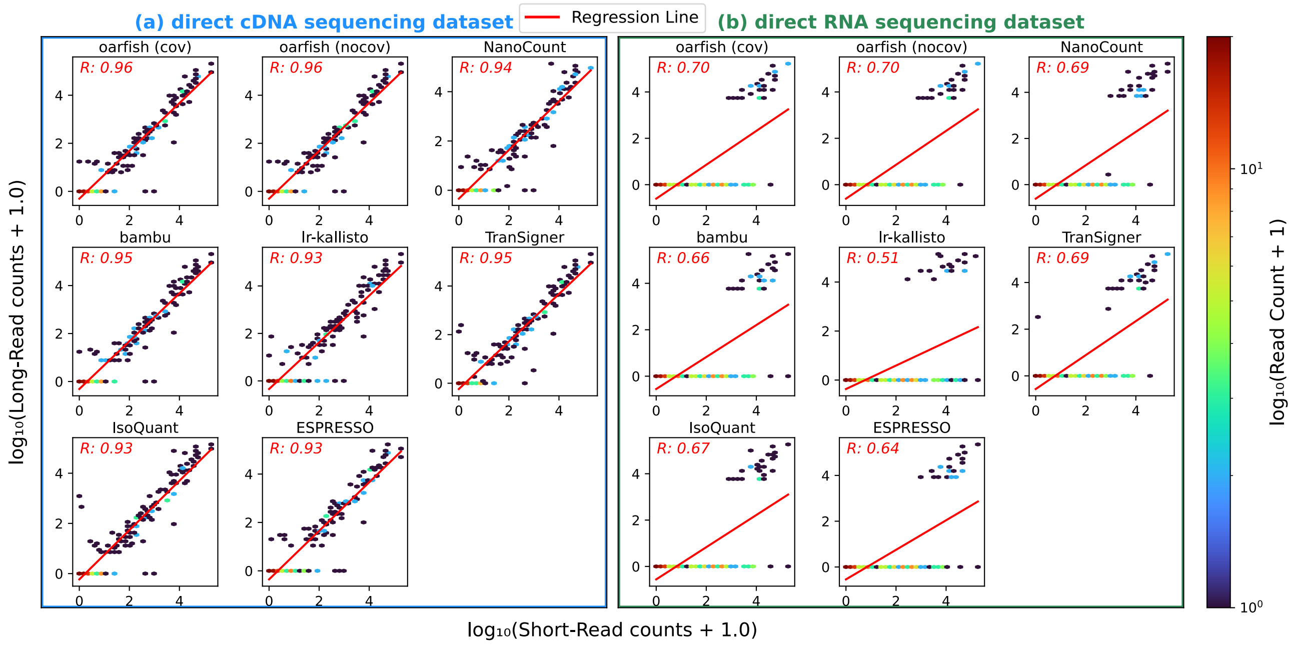

Supplementary Figure S21. Density plot for long read counts obtained from the **Hct116 cell line** and their corresponding known concentration on the sequin transcripts. In all of these methods, the p-value for the Pearson correlation is almost zero (P-value $\simeq 0.0$). (a) The ONT long read RNA-seq dataset sequenced with `direct-cDNA` protocol. (b) The ONT long read RNA-seq dataset sequenced with `direct-RNA` protocol.

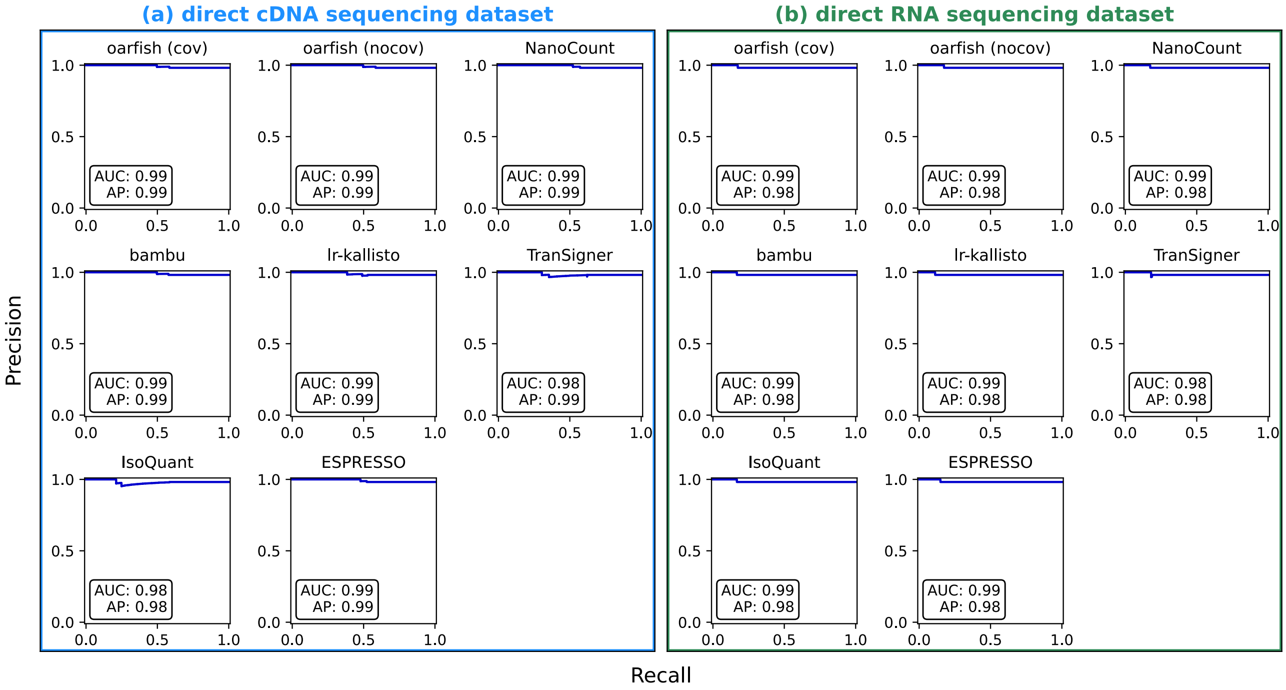

Supplementary Figure S22. precision-recall curve for long read counts obtained from the **Hct116 cell line** and their corresponding known concentration on sequin transcripts. (a) The ONT long read RNA-seq dataset sequenced with `direct-cDNA` protocol. (b) The ONT long read RNA-seq dataset sequenced with `direct-RNA` protocol.

H.5 Experimental Dataset (SH-SY5Y): Correlation & Error Metrics

The experimental dataset derived from the SH-SY5Y cell line includes 1D cDNA and direct-RNA sequencing data, along with TEQUILA-seq data from both 4-hour and 8-hour sequencing runs. As illustrated in Figure S23 and Figure S24, `oarfish` (both with and without the coverage model) consistently outperforms other methods across most metrics and sequencing protocols—1D cDNA, direct-RNA, TEQUILA-seq 4h, and TEQUILA-seq 8h. This trend is especially prominent for transcripts with lower InfRV values and higher certainty in their short-read counts. Although there are minor deviations, such as slightly higher RMSE and NRMSE in the 1D cDNA dataset, Pearson and CCC correlations for the direct-RNA dataset, and MARD in the TEQUILA-seq 8h dataset, these differences are marginal (within 1%). Thus, we conclude that `oarfish` consistently delivers superior performance compared to other methods across these ONT sequencing datasets, particularly for targeted transcripts in the TEQUILA-seq protocol.

The SH-SY5Y dataset includes SIRV spike-in transcripts, providing an additional benchmark. While all methods demonstrate comparable performance across the various metrics due to the limited number of SIRV transcripts (46 in total), `oarfish` remains one of the top-performing methods. Furthermore, Figure S25 highlights nearly equivalent performance across methods and protocols for paired metrics, with the exception of the TEQUILA-seq 8h dataset, where `IsoQuant` shows a slight edge. Beyond this, given the small number of targeted spike-in transcripts, all methods show closely aligned performance in both the strength and direction of the linear relationship between long-read counts and known concentrations (Figure S26) and in distinguishing between expressed and non-expressed transcripts (Figure S27).

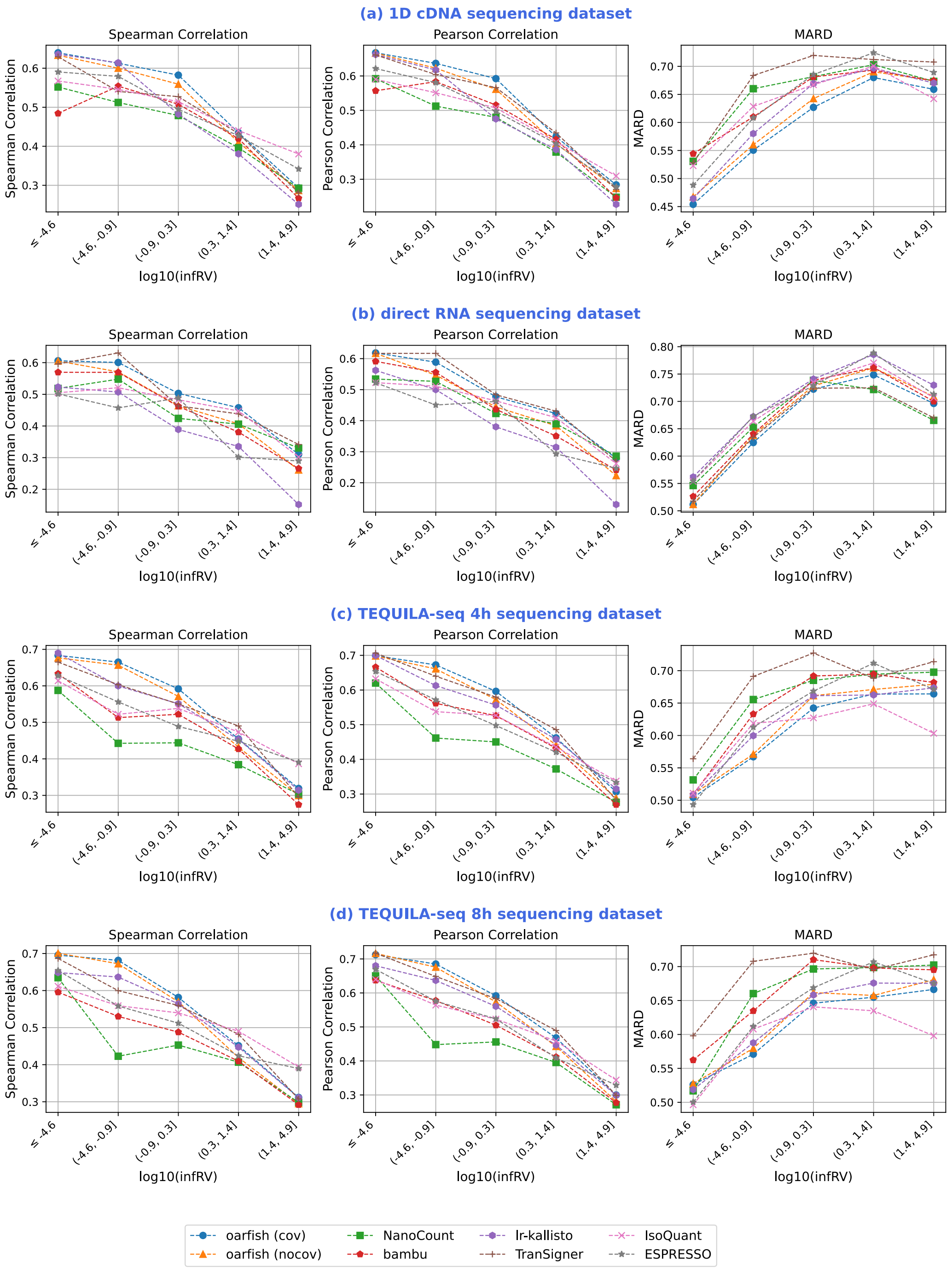

Supplementary Figure S23. Spearman correlation, Pearson correlation, and MARD between long read and short read quantification results on different subset of InfRV values on all the transcripts for **SH-SY5Y cell line** dataset. (a) The correlation and error metrics for ONT long read RNA-seq dataset sequenced with `1D-cDNA` protocol. (b) The correlation and error metrics for ONT long read RNA-seq dataset sequenced with `direct-RNA` protocol. (c) The correlation and error metrics for ONT long read RNA-seq dataset sequenced with `TEQUILA-seq 4 hour sequencing` protocol. (d) The correlation and error metrics for ONT long read RNA-seq dataset sequenced with `TEQUILA-seq 8 hour sequencing` protocol.

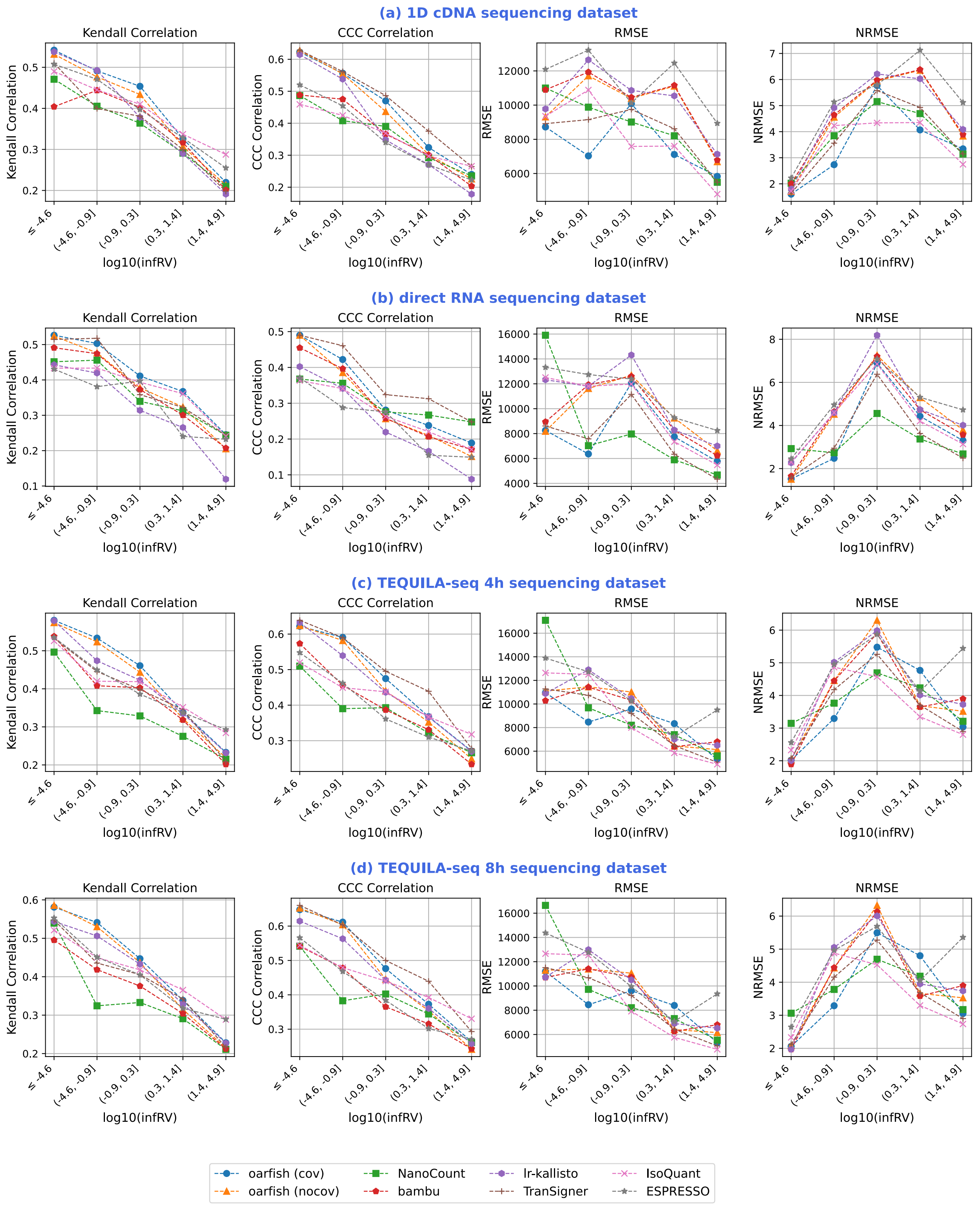

Supplementary Figure S24. Kendall correlation, CCC correlation, RMSE, and NRMSE between long read and short read quantification results on different subset of InfRV values on all the transcripts for **SH-SY5Y cell line** dataset. (a) The correlation and error metrics for ONT long read RNA-seq dataset sequenced with `1D-cDNA` protocol. (b) The correlation and error metrics for ONT long read RNA-seq dataset sequenced with `direct-RNA` protocol. (c) The correlation and error metrics for ONT long read RNA-seq dataset sequenced with `TEQUILA-seq 4 hour sequencing` protocol. (d) The correlation and error metrics for ONT long read RNA-seq dataset sequenced with `TEQUILA-seq 8 hour sequencing` protocol.

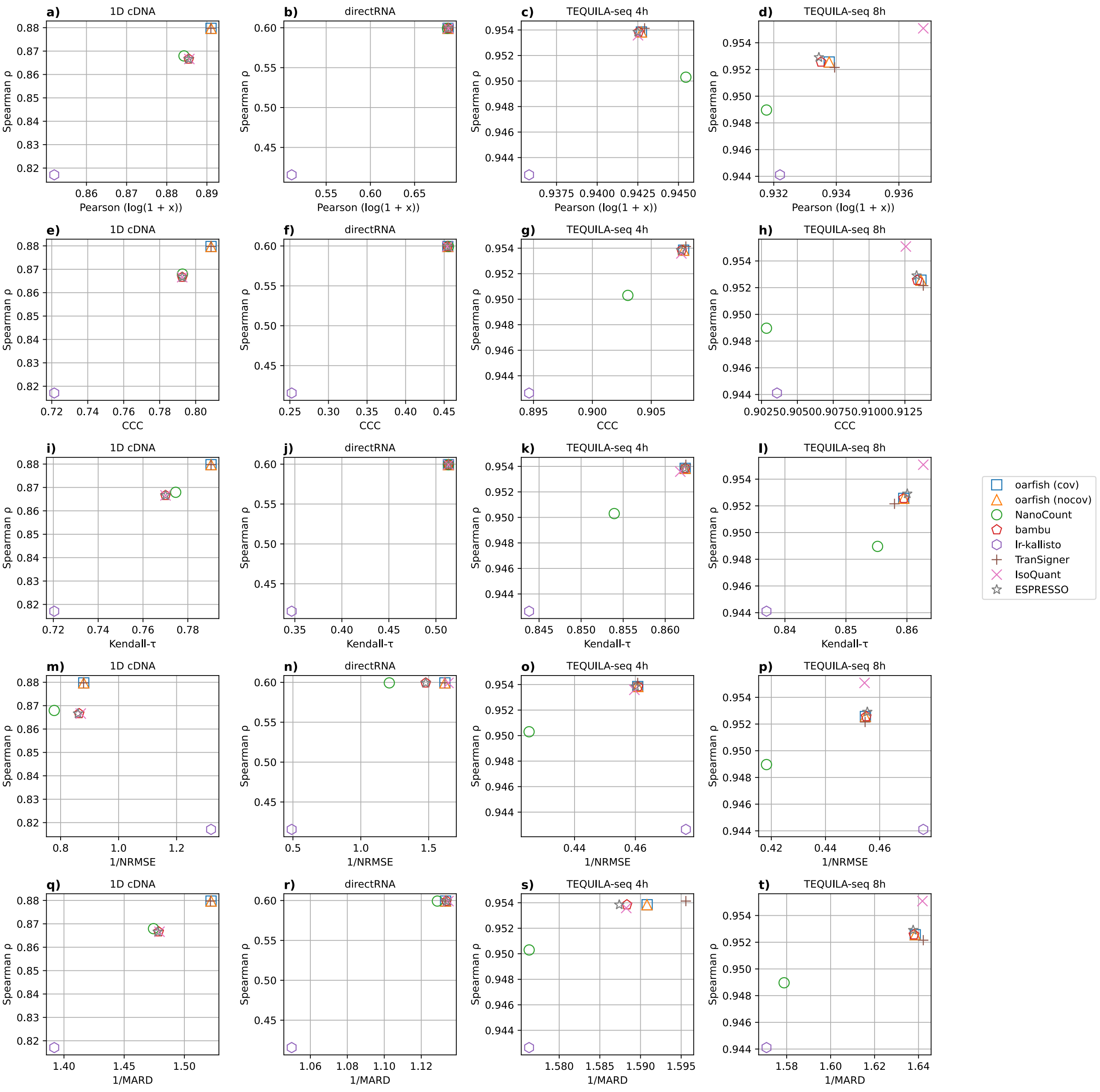

Supplementary Figure S25. compare metrics for `1D-cDNA`, `direct-RNA`, `TEQUILA-seq 4h`, and `TEQUILA-seq 8h` datasets sequenced from **SH-SY5Y cell line** sample.

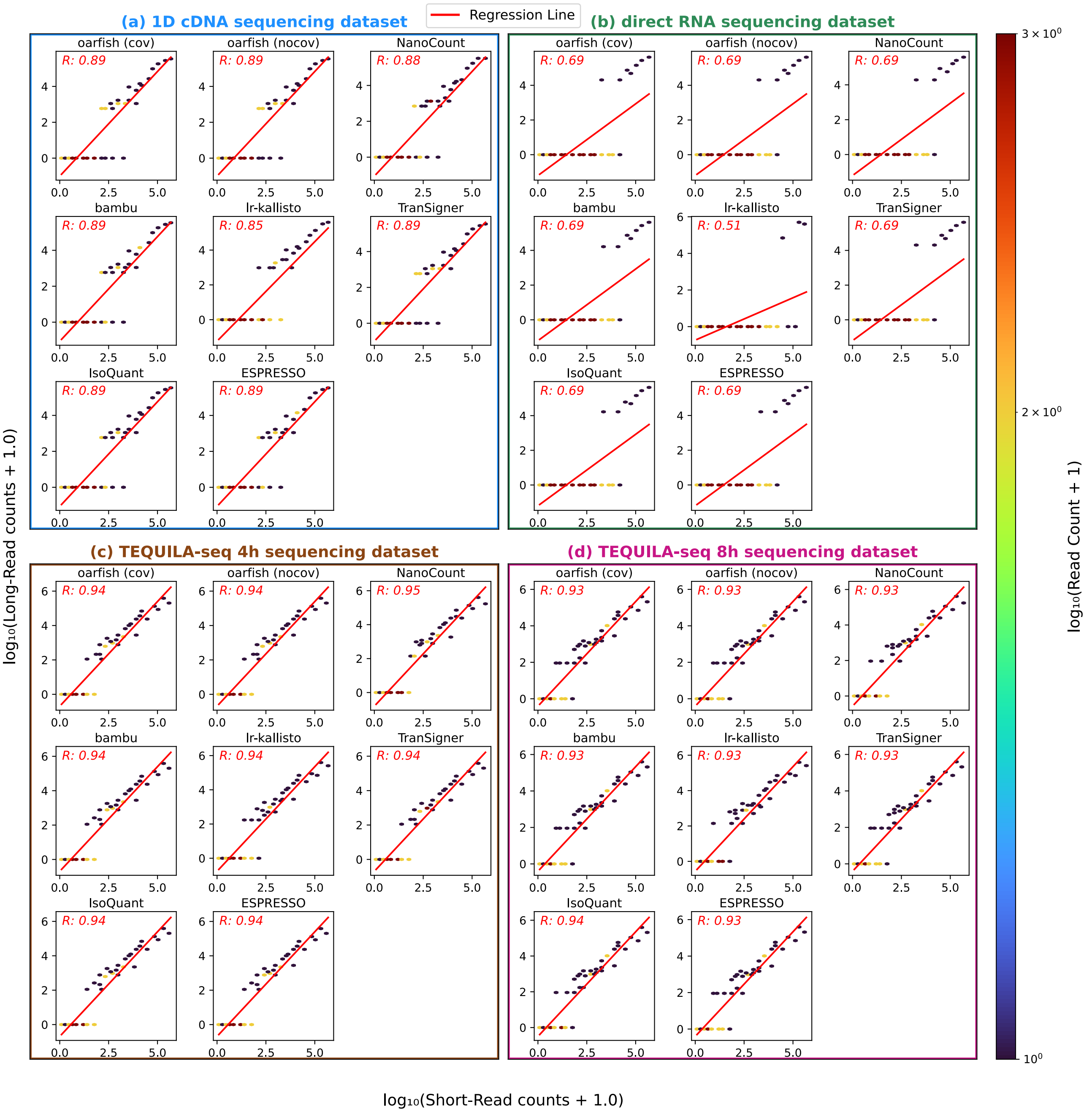

Supplementary Figure S26. Density plot for long read counts obtained from the **SH-SY5Y cell line** and their corresponding known concentration on the SIRV transcripts. In all of these methods, the p-value for the Pearson correlation is almost zero (P-value ≃0.0). (a) The ONT long read RNA-seq dataset sequenced with `1D-cDNA` protocol. (b) The ONT long read RNA-seq dataset sequenced with `direct-RNA` protocol. (c) The ONT long read RNA-seq dataset sequenced with `TEQUILA-seq 4h` protocol. (d) The ONT long read RNA-seq dataset sequenced with `TEQUILA-seq 8h` protocol.

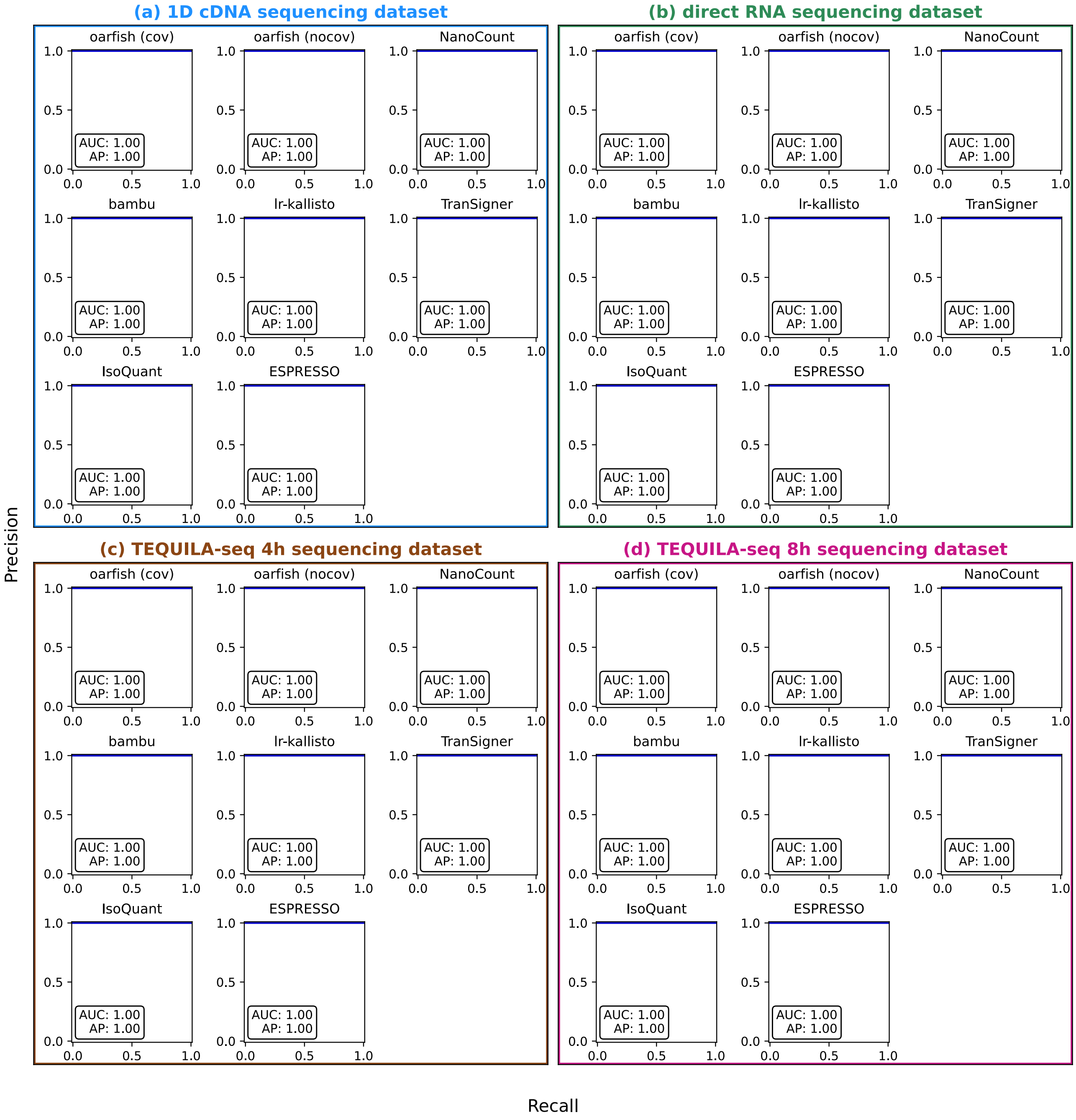

Supplementary Figure S27. precision-recall curve for long read counts obtained from SH-**SY5Y cell line** and their corresponding known concentration on SIRV transcripts. (a) The ONT long read RNA-seq dataset sequenced with `1D-cDNA` protocol. (b) The ONT long read RNA-seq dataset sequenced with `direct-RNA` protocol. (c) The ONT long read RNA-seq dataset sequenced with `TEQUILA-seq 4 hour sequencing` protocol. (d) The ONT long read RNA-seq dataset sequenced with `TEQUILA-seq 8 hour sequencing` protocol.

H.6 Experimetnal Datasets (Hct116, UHRR, SH-SY5Y): Runtime & memory usage

We evaluated the time and memory efficiency of our proposed method alongside several leading tools on experimental datasets from ONT and PacBio platforms. Significant differences in resource usage were observed across the tools, with `ESPRESSO` being particularly resource-intensive, requiring 2 to 13 hours of runtime and 110 to 140 GB of memory. Since `ESPRESSO` performs both identification and quantification, and unlike `bambu`, its identification step cannot be disabled, we excluded `ESPRESSO` from our time and memory usage analysis to ensure a fair comparison among quantification-only tools.

As shown in figs. S28 and S30, all methods, except for `bambu` and `IsoQuant`, exhibited similar runtimes across datasets from the Hct116 and SH-SY5Y cell lines. In fig. S29, `lr-kallisto`, a pseudoalignment-based method, showed a slight improvement in runtime over alignment-based methods such as `oarfish`, `NanoCount`, and `TranSigner` when processing the UHRR dataset, which is considerably larger than the Hct116 and SH-SY5Y datasets. However, in terms of memory usage, `oarfish` outperformed all other tools, including `lr-kallisto`, which was expected to have the lowest resource consumption due to its pseudoalignment approach.

In conclusion, `oarfish` consistently demonstrated excellent resource efficiency, comparable to the lightweight pseudoalignment approach of `lr-kallisto`.

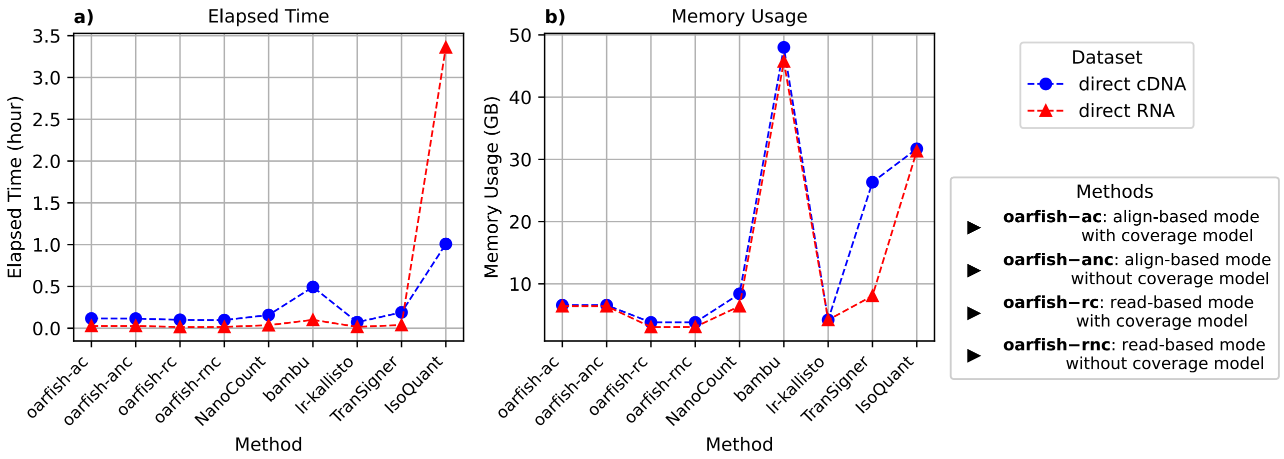

Supplementary Figure S28. Performance metrics (elapsed time and peak memory usage) for the proposed and alternative methods using the `direct-cDNA` and `direct-RNA` experimental datasets from the **Hct116 cell line**: (a) Elapsed time for all methods presented in hours. (b) Memory usage for all methods displayed in Gigabytes. In these figures, `direct-cDNA` and `direct-RNA` represent ONT long reads sequenced with `direct-cDNA` and `direct-RNA` protocols, respectively.

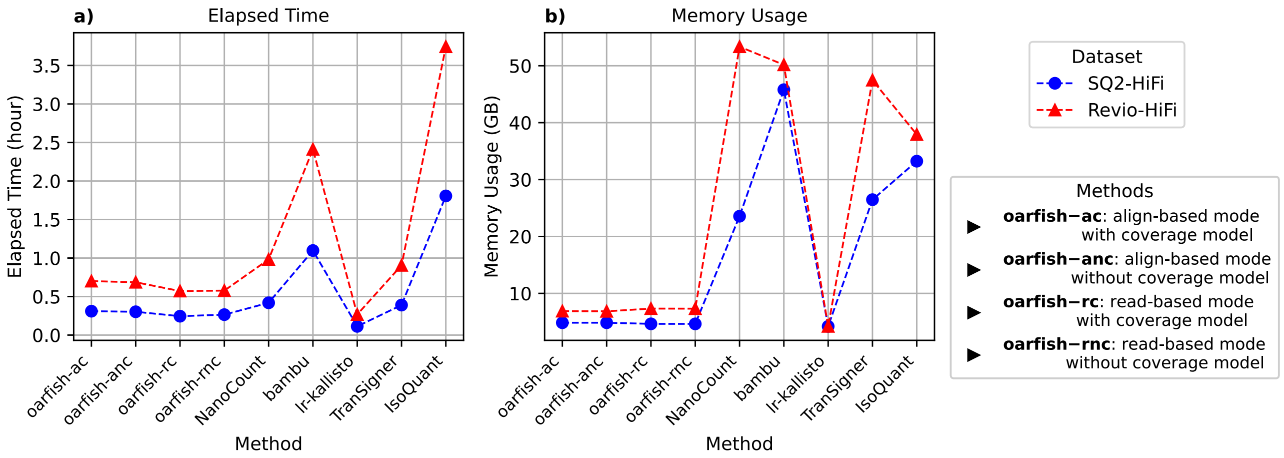

Supplementary Figure S29. Performance metrics (elapsed time and peak memory usage) for the proposed and alternative methods using the `SQ2-HiFi` and `Revio-HiFi` experimental datasets from the **UHRR sample**: (a) Elapsed time for all methods presented in hours. (b) Memory usage for all methods displayed in Gigabytes. In these figures, `SQ2` and `Revio` represent PacBio long reads sequenced with `SQ2` and `Revio` protocols, respectively.

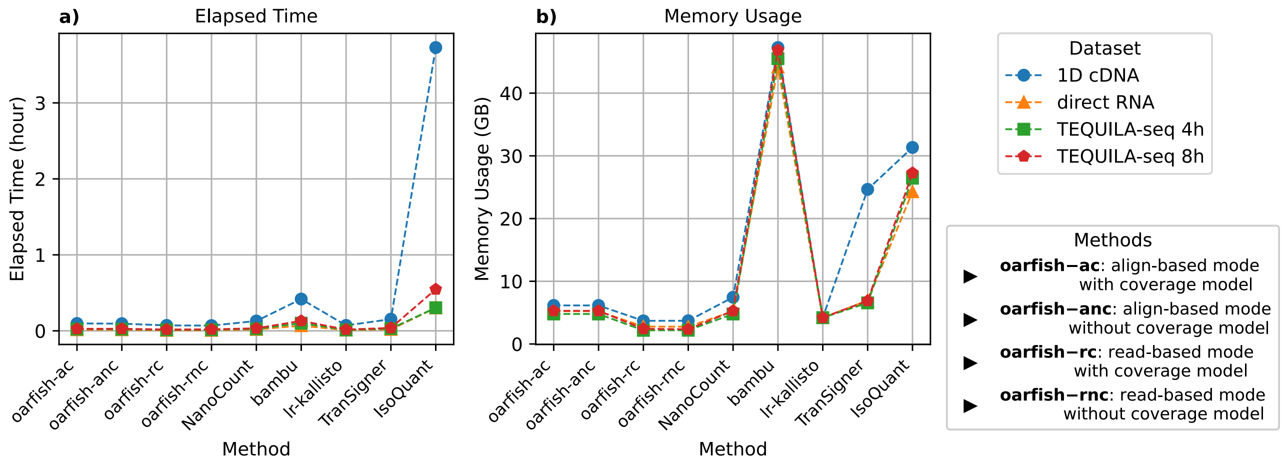

Supplementary Figure S30. Performance metrics (elapsed time and peak memory usage) for the proposed and alternative methods using the `1D-cDNA`, `direct-RNA`, `TEQUILA-seq 4h`, and `TEQUILA-seq 8h` experimental datasets from the **SH-SY5Y cell line**: (a) Elapsed time for all methods presented in hours. (b) Memory usage for all methods displayed in Gigabytes. In these figures, `1D cDNA`, `direct-RNA`, `TEQUILA-seq 4h`, and `TEQUILA-seq 8h`, represent ONT long reads sequenced with `1D-cDNA`, `direct-RNA`, `TEQUILA-seq 4h` and `TEQUILA-seq 8h` protocols, respectively.
